# Dual‐Antenna Trimetallic Lanthanide Complexes for Enhanced Near‐Infrared Luminescence

**DOI:** 10.1002/asia.202500017

**Published:** 2025-06-17

**Authors:** Krishanu Bandyopadhyay, Abhineet Verma, Satyen Saha

**Affiliations:** ^1^ Department of Chemistry Institute of Science Banaras Hindu University Varanasi 221005 India; ^2^ Department of Chemistry Malaviya National Institute of Technology (MNIT) Jaipur 302017 India

**Keywords:** Antenna effect, Enegry transfer efficiency, Enhanced luminescence, Lanthanide luminescence, NIR emitting materials, Photophysics of lanthanide complexes

## Abstract

Lanthanide [Ln(III)] ions are known for their unique near‐infrared (NIR) luminescence, typically achieved through indirect excitation via the antenna effect. Organic ligands, such as *N*,*N*‐bis(3‐methoxysalicylidene)‐1,4‐diamino butane (**L**), in combination with Zn, have previously demonstrated their effectiveness in enhancing lanthanide NIR emission, as seen in bimetallic [**L‐Zn‐Ln**] complexes, which employs a single antenna. In this study, we present a new series of trimetallic Zn–Ln complexes, [**(L‐Zn)₂‐Ln**], featuring two compartmental ligand‐Zn complexes, acting as antennas, aimed at further improving energy transfer efficiency to the lanthanide centers. Comprehensive characterization using SCXRD, PXRD, FT‐IR, and CHN analyses confirmed the structural integrity of these complexes. Notably, SCXRD and XPS revealed significant structural differences between the bimetallic and trimetallic systems. The impact of the additional antenna, replacing nitrate and methanol—known contributors to nonradiative relaxation in the bimetallic [**L‐Zn‐Ln**] complexes—was thoroughly examined. Photophysical studies across both visible and NIR regions demonstrated substantial enhancements in luminescence, particularly in the NIR region, attributed to the inclusion of the second antenna, highlighting its role in improving the overall energy transfer process.

## Graphical abstract

Dual‐antenna trimetallic lanthanide complexes, [(L‐Zn)_2_‐Ln], exhibit enhanced near‐infrared luminescence through improved energy transfer from two Zn‐ligand antenna units. Compared to bimetallic analogues, these complexes show reduced non‐radiative losses and superior NIR emission. Structural and spectroscopic studies confirm the role of dual antennas in boosting luminescence efficiency, highlighting their potential in NIR‐based photonic applications.

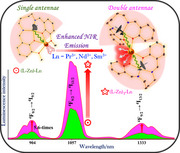

## 1 Introduction

Near‐infrared (NIR)‐emitting lanthanide complexes have garnered significant attention due to their intriguing optical,^[^ ^1^ ^]^ magnetic,^[^ ^2^ ^]^ electrical,^[^ ^3^ ^]^ and catalytic properties,^[^ ^4^ ^]^ making them promising candidates for biological imaging,^[^ ^5^ ^]^ materials science,^[^ ^6^ ^]^ solar energy conversion,^[^ ^7^ ^]^ LED technology,^[^ ^8^ ^]^ sensing,^[^ ^9^ ^]^ and time‐resolved immune testing.^[^ ^10^ ^]^ These complexes are particularly appealing because of the narrow bandwidth emission, high resistance to photobleaching, and stable emission band positions of Ln(III) ions, regardless of the surrounding environment.^[^ ^11^ ^]^

Recent research has focused heavily on NIR‐emitting Ln‐MOFs, particularly those based on Yb(III), Nd(III), and Er(III). However, the intrinsic low molar extinction coefficient (<10 M^−1^ cm^−1^) of lanthanide ions often results in poor luminescence intensity.^[^ ^12^ ^]^ Nonradiative relaxation processes, primarily caused by N─H, O─H, or C─H oscillators in organic ligands or coordinated solvents, can significantly quench NIR emission.^[^ ^13^ ^]^ To improve luminescence quantum yields, shielding lanthanide ions from these oscillators by removing coordinated solvents has proven effective.^[^ ^14^, ^15^, ^16^ ^]^

Various strategies have emerged in the literature to tackle this challenge.^[^ ^17^, ^18^, ^19^, ^20^, ^21^, ^22^, ^23^, ^24^, ^25^, ^26^, ^27^, ^28^, ^29^, ^30^, ^31^ ^]^ One method involves employing deuterated or halogenated organic ligands to mitigate the quenching effects from C─H vibrations, though this often entails multiple synthetic steps.^[^ ^17^, ^18^, ^19^ ^]^ Another promising approach is the design of rigid ligands with extensive π–π conjugation systems.^[^ ^20^, ^21^ ^]^ These reduce intramolecular vibrations, preventing energy dissipation as heat.^[^ ^22^, ^23^ ^]^ Additionally, the bulky nature of these ligands can effectively shield Ln(III) ions from external environments, reducing quenching by solvent molecules.^[^ ^23^ ^]^ Lanthanide complexes with large conjugated ligands, particularly those emitting in the NIR range, are especially suitable for biochemistry and cellular biology.^[^ ^21^ ^]^ This is because shifting the absorption from the UV to the visible range minimizes tissue damage during UV exposure.^[^ ^20^ ^]^

Another strategy involves employing 3d–4f heteronuclear lanthanide platforms, which lower the triplet state of ligands and enhance luminescence.^[^ ^24^, ^25^, ^26^, ^27^ ^]^ These complexes are also relatively straightforward to synthesize, with 3d metal ions providing benefits such as low excitation energy, long luminescence lifetimes, and high quantum yield.^[^ ^25^ ^]^ In these systems, organic ligands paired with 3d‐metal ions function as antennas, absorbing energy and efficiently transferring it to Ln(III) ions, which are otherwise challenging to excite directly.^[^ ^26^ ^]^ The choice of antenna is pivotal, as the efficiency of energy transfer hinges on the alignment between the ligand's triplet state and the lowest excited state of the Ln(III) ions.^[^ ^25^ ^]^

Various d‐block ions, including Ru(II), Os(II), Pt(II), Pd(II), and Zn(II), have been utilized to form d‐f bimetallic complexes. Among them, Zn(II) plays a key role due to its ability to stabilize the structure within 3d–4f complexes and enable sensitized NIR luminescence from Ln(III).^[^ ^28^, ^29^, ^30^ ^]^ In our previous studies, Zn was selected for its d¹⁰ electronic configuration, which has proven more efficient as an antenna than other d‐block ions like Cu(II) and Ni(II).^[^ ^31^ ^]^ Schiff base ligands, in combination with Zn, are excellent chromophores for visible and NIR luminescence, leading to the development of numerous Zn–Ln complexes with varying nuclearities, including dinuclear, trinuclear, and tetranuclear forms.^[^ ^32^, ^33^, ^34^, ^35^, ^36^, ^37^, ^38^ ^]^

Enhancing the NIR emission from NIR‐emitting materials has always been challenging for researchers. Various approaches have been adopted to get enhancement of the NIR emission.^[^ ^39^, ^40^, ^41^ ^]^ Klonkowski et al. and Zhang et al. improved the emission intensity of luminescent material by encapsulating the complex in silica or methylated silicate xerogels.^[^ ^39^, ^40^ ^]^ Whereas Nguyen et al. used sterically hindered ligands to increase the luminescence intensity, which isolate the lanthanide from the coordinating solvent.^[^ ^41^ ^]^ Peralta et al. discussed luminescent properties of NTB and BBP ligands in Ln(III) complexes depend on ligand structure, coordination symmetry, and functionalization. While BBP ligands show better energy transfer and luminescence efficiency, NTB complexes with specific substituents or low‐symmetry environments can also enhance quantum yields. These complexes hold significant potential for applications in advanced materials, cancer therapies, and biological imaging.^[^ ^41^ ^]^

In this study, we propose a straightforward and cost‐effective synthesis method to enhance luminescent intensity in the NIR region. Our earlier research on bimetallic [(**L‐Zn**)**‐Ln**] complexes revealed the coordination of nitrate ions and methanol, along with the antenna ligand. These induced nonradiative relaxation processes reduced NIR luminescence intensity.^[^ ^42^ ^]^ To address this issue, we have advanced the bimetallic [**(L‐Zn)‐Ln**] system into a trimetallic [**(L‐Zn)₂‐Ln**] configuration. By introducing two antenna ligands and eliminating nitrate and methanol, aim to improve energy transfer to the excited states of lanthanides, thereby increasing NIR luminescence intensity.

## 2 Methodology

### 2.1 Design and Synthesis of [(L‐Zn)─Ln] and [(L‐Zn)_2_‐Ln]

The organic ligand (**L**: *N,N*‐bis(3‐methoxysalicylidene)‐1,4‐diaminobutane) was synthesized following a procedure previously reported by our group.^[^ ^31^, ^42^ ^]^ In brief, 1,4‐diaminobutane (1 mmol, 0.088 g) was reacted with 3‐methoxysalicylaldehyde (2 mmol, 0.304 g) under reflux in ethanol for 3 h. The resulting light‐yellow precipitate was collected, washed with hexane and cold ethanol, and dried under a high vacuum. The ligand was fully characterized by ^1^H NMR (Supporting Information Figure S1).

**Scheme 1:**
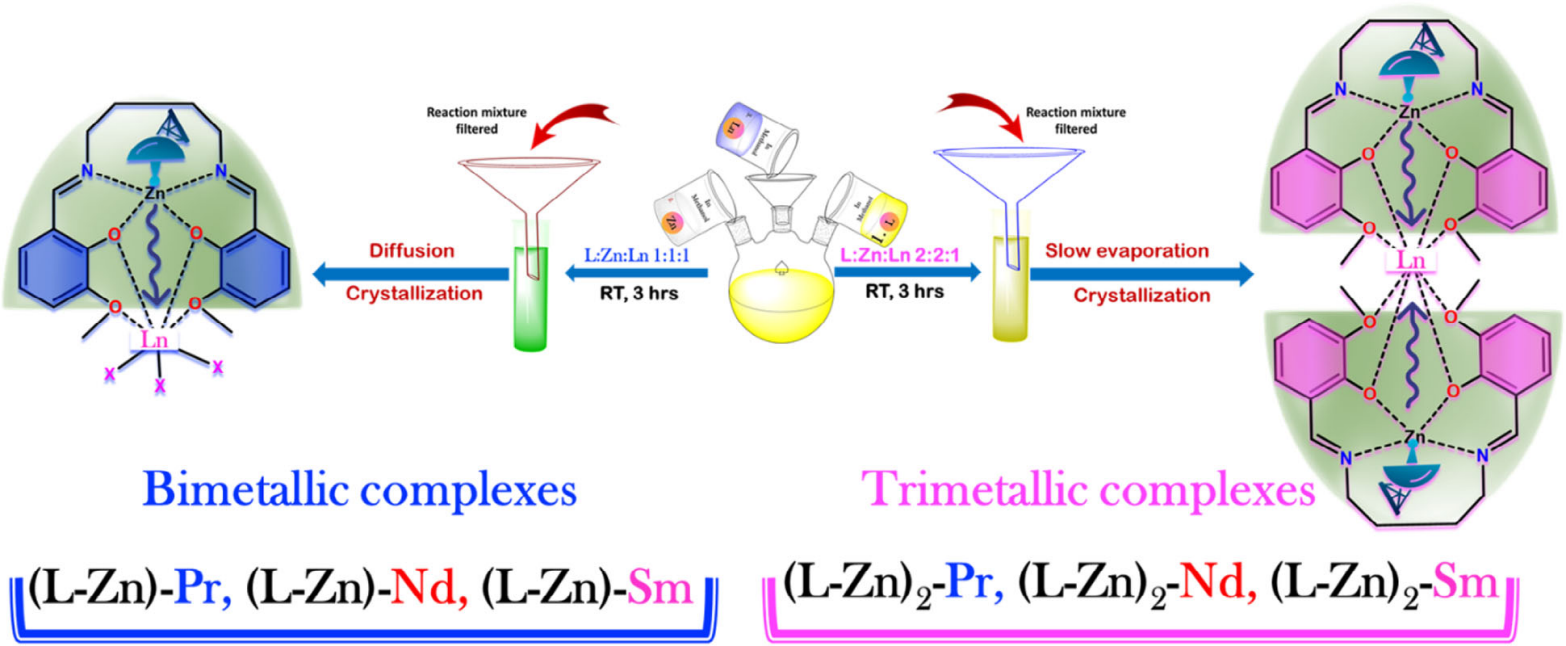
Synthetic route for the preparation of the bimetallic (X stands for nitrate and methanol)^[^ ^42^ ^]^ and trimetallic complexes.

Bimetallic complexes [(**L‐Zn)‐Ln**] were synthesized following our previously established procedure (Scheme 1).^[^ ^42^ ^]^ These complexes were prepared in situ stepwise, without isolating the mononuclear ligand‐d‐block metal intermediates. Building on this approach, the new trimetallic complexes [**(L‐Zn)₂‐Ln**] were designed and synthesized with a slight modification to the original method. By adjusting the reactant ratio from L:Zn:Ln (1:1:1) to (2:2:1), dual‐antenna trimetallic complexes were obtained, again without the isolation of mononuclear intermediates. Although all trinuclear complexes were synthesized using the similar methods, here only the detailed synthesis of **[(L‐Zn)₂‐Pr**] is presented, with the analytical data for the rest of the complexes.

[(**L‐Zn)_2_‐Pr**]: 20 mL of a methanolic solution of **L** (0.71 g, 2.0 mmol) was added to 15 mL of a methanolic solution of Zn‐(CH_3_COO)_2_·2H_2_O (0.44 g, 2.0 mmol) while stirring, followed by dropwise addition of a 15 mL methanolic solution of Pr(NO_3_)_3_·6H_2_O (0.43 g, 1.0 mmol). The reaction mixture was kept stirring for 3 h. The reaction mixture was then cooled to RT and filtered to eliminate any unreacted starting materials. The filtrate was kept for crystallization using a slow evaporation method under a controlled environment (having 15 to 20 °C). Light yellow needle‐shaped crystals for [**Pr**] suitable for single crystals for SCXRD measurements were obtained within a week. The time taken for crystallization varies with the systems, spanning up to 7 days. Yield: 0.91 g (1.1 mmol, 90.1%); M.P.: 209 (±2) °C; Anal. calcd for C_44_H_50_N_5_O_15_Zn_2_Pr (M.W. = 1160.61): C 45.53; H 4.34; N 6.03. Found C 45.63; H 4.63; N 6.23.

**[(L‐Zn)_2_Nd**]: 0.89 g (1.2 mmol, 89.5%; M.P. 203 (±2)°C; Anal. calcd for C_44_H_50_N_5_O_15_Zn_2_Nd (M.W. = 1163.94): C 45.40; H 4.33; N 6.02. Found C 45.92; H 4.46; N 6.13.

**[(L‐Zn)_2_Sm**]: 0.95 g (1.3 mmol, 92.5%); M.P. 208 (±2)°C; Anal. calcd for C_44_H_50_N_5_O_15_Zn_2_Sm (M.W. = 1170.07): C 45.16; H 4.31; N 5.99. Found C 45.23; H 4.41; N 5.92.

## 3 Result and Discussion

### 3.1 Structural Studies of [(L‐Zn)‐Ln] and [(L‐Zn)_2_‐Ln] Complexes

The synthesis of bimetallic and trimetallic complexes incorporating ligand (**L**) was conducted stepwise in situ, without isolating any intermediate Zn or Ln–Zn complexes. The resulting complexes were characterized by utilizing FT‐IR, PXRD, and SCXRD techniques. FT‐IR spectra revealed a prominent peak between 1615 and 1630 cm^−1^ across all six complexes, corresponding to the formation of the azomethine (C═N) group in the Schiff base.^[^ ^43^, ^44^, ^45^ ^]^ The occurrence of complexation was further evidenced by a blue shift in the phenolic C─O stretching band from 1214 cm^−1^ in the ligand to 1249 cm^−1^ upon complex formation, indicating deprotonation followed by coordination of the phenolic oxygen donor to the metal center.^[^ ^46^ ^]^ A vibrational mode at 920 cm^−1^ was assigned to the C─C stretching in the ─(O)₂C─CH₃ group. (shown in Supporting Information Figure S2) The counter anions exhibited their characteristic vibrational bands in the FT‐IR spectra, with a mode around 1469 cm^−1^ confirming the presence of the acetate group.^[^ ^36^ ^]^ Sharp bands observed at 570 cm^−1^ corresponded to M─N (nitrate group) stretching frequencies in the bimetallic complexes; these bands were absent in the trimetallic complexes due to substituting the nitrate counterpart with another Zn–Ln antenna.^[^ ^47^ ^]^ Additionally, a stretching frequency at 446 cm^−1^ was detected in both series of complexes, attributable to the presence of an M─O bond within the acetate group residue. FT‐IR spectra of all the complexes are given in the Supporting Information (Figure S2).

The bulk purity of the complexes was confirmed through ^1^H NMR, PXRD patterns, HRMS, and CHN analysis, as shown in Supporting Information Figure S1, Figure S3, and Figure S4, respectively. The experimental PXRD patterns for both **[(L‐Zn)‐Ln**] and [(**L‐Zn)_2_‐Ln**] complexes closely matched the simulated patterns derived from SCXRD data, confirming that the crystal structures accurately represent the bulk material.

### 3.2 Structural Analysis by SCXRD

**Figure 1:**
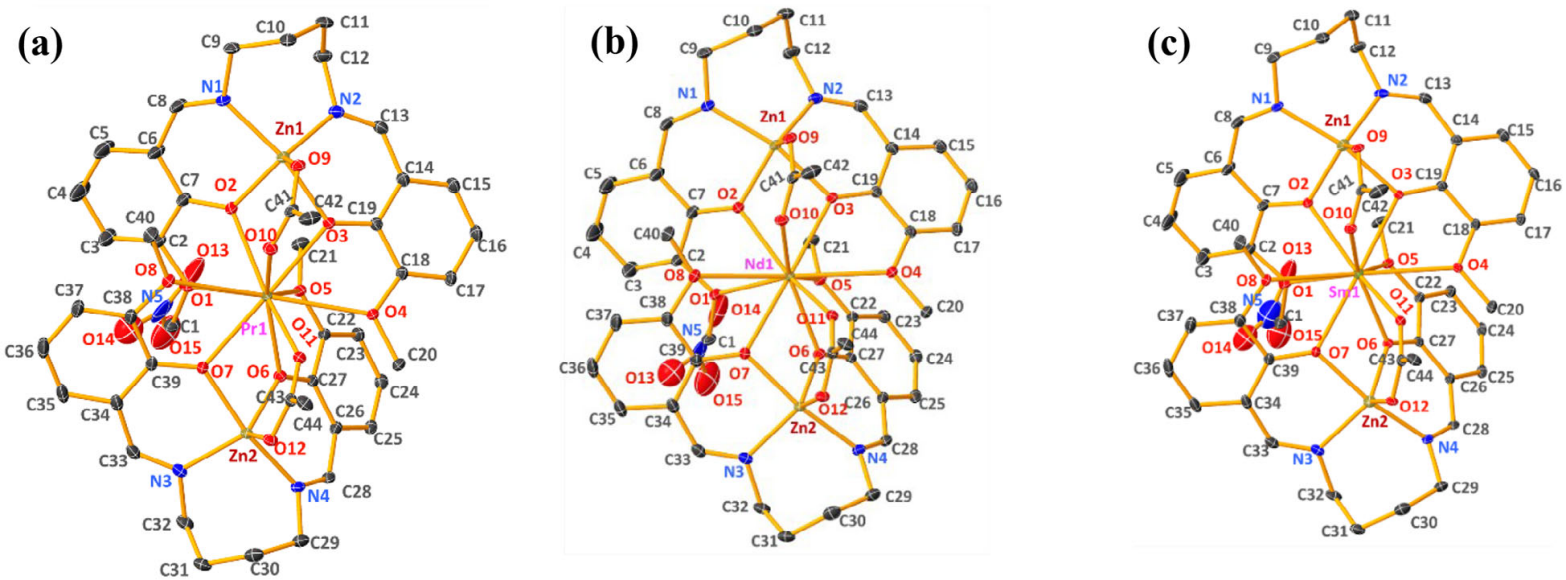
ORTEP diagram of (a) [(**L‐Zn)_2_‐Pr**], (b) [(**L‐Zn)_2_‐Nd**], and (c) [(**L‐Zn)_2_‐Sm**] complexes presented at 30% probability ellipsoid.

All the complexes were fully characterized by SCXRD, and the key crystal data for all the complexes are listed in Table 1, while significant bond angles and lengths are detailed in Table 2. All the bimetallic **[(L‐Zn)‐Ln]** complexes (previously presented)^[^ ^42^ ^]^ are isostructural and crystallize in a monoclinic crystal system with *P2_1_/c* space group, whereas the trimetallic **[(L‐Zn)_2_‐Ln**] crystallize in a triclinic crystal system with $P\bar{1}$ space group. SCXRD measurements for trimetallic complexes were performed at 100 K due to high thermal ellipsoids and dynamic disorder observed at 298 K, which compromised data quality. In contrast, bimetallic complexes were recorded at RT yielded high‐quality data. The ORTEP diagrams for all the trimetallic complexes are shown in Figure 1.

**Table 1:** Important crystal data for **[(L‐Zn)−Ln]** and **[(L‐Zn)_2_**‐**Ln]** complexes.

Compounds	[(L‐Zn)‐Pr]^[^ ^42^ ^]^	[(L‐Zn)‐Nd]^[^ ^42^ ^]^	[(L‐Zn)‐Sm]^[^ ^42^ ^]^	[(L‐Zn)_2_‐Pr]	[(L‐Zn)_2_‐Nd]	[(L‐Zn)_2_‐Sm]
Empirical formula	C_23_H_29_N_4_O_13_ZnPr	C_23_H_29_N_4_O_13_ZnNd	C_23_H_29_N_4_O_13_ZnSm	C_44_H_50_N_5_O_15_Zn_2_Pr	C_44_H_50_N_5_O_15_Zn_2_Nd	C_44_H_50_N_5_O_15_Zn_2_Sm
**CCDC No**.	1480672	1480671	1480673	2345778	2345777	2345779
F.W.	774.77	792.15	784.24	1160.61	1163.94	1170.07
Space group	*P2_1_/c*	*P2_1_/c*	*P2_1_/c*	$P\bar{1}$	$P\bar{1}$	$P\bar{1}$
Crystal system	monoclinic	monoclinic	monoclinic	triclinic	triclinic	triclicnic
*a* (Å)	12.110(5)	12.014(5)	11.436(5)	12.0542(4)	12.0542(4)	12.0142(4)
*b* (Å)	23.772(5)	23.693(5)	23.800(5)	15.0150(3)	14.9850(3)	14.9950(3)
*c* (Å)	11.489(5)	11.509(5)	11.895(5)	15.2744(4)	15.2008(4)	15.1244(4)
*α* (°)	90	90	90	84.867(2)	85.0120(19)	85.1667(19)
*β* (°)	112.00(5)	111.88(5)	111.80(5)	74.860(2)	75.077(3)	75.660(3)
*γ* (°)	90	90	90	72.940(2)	72.947(2)	72.940(2)
*V* (Å^3^)	3060.0(2)	3039.9(19)	3005.9(2)	2550.94(12)	2536.31(12)	2523.46
*T* (K)	293	293	293	100.15	100.15	100.15
*Z*	4	4	4	2	2	2
*D_calc_* (mg m^−3^)	1.682	1.700	1.733	1.511	1.524	1.540
*μ* (mm^−1^)	2.424	2.545	2.800	8.876	9.367	2.160
GOF on *F^2^*	1.220	1.049	1.166	1.025	1.026	1.024
*R (F_o_^2^)* ^a)^	0.0663	0.0809	0.1067	0.0366	0.0376	0.0569
*Rw (F_o_^2^)* ^b)^	0.1167	0.1809	0.1944	0.0389	0.1002	0.1328

^a)^ *R1* = Σ||*F*_o_|−|*F*_c_||/Σ|*F*_o_|.

^b)^ *wR2* = [Σ[*w*(*F*_o_^2^−*F*_c_^2^)^2^]/Σ[*w*(*F*_o_^2^)^2^]^1/2^.

**Table 2:** Important bond angles (^○^), bond lengths (Å), and dihedral angles (^○^) for **[(L‐Zn)‐Ln]** and **[(L‐Zn)_2_‐Ln]** complexes. Pictorial depictions of the bond angles and lengths are given below the tabulated data.

Complex	(L‐Zn)_2_‐Pr		(L‐Zn)‐Pr	(L‐Zn)_2_‐Nd		(L‐Zn)‐Nd	(L‐Zn)_2_‐Sm		(L‐Zn)‐Sm	
**Angle (°)**	**Zn1**	**Zn2**	**Zn**	**Zn1**	**Zn2**	**Zn**	**Zn1**	**Zn2**	**Zn**	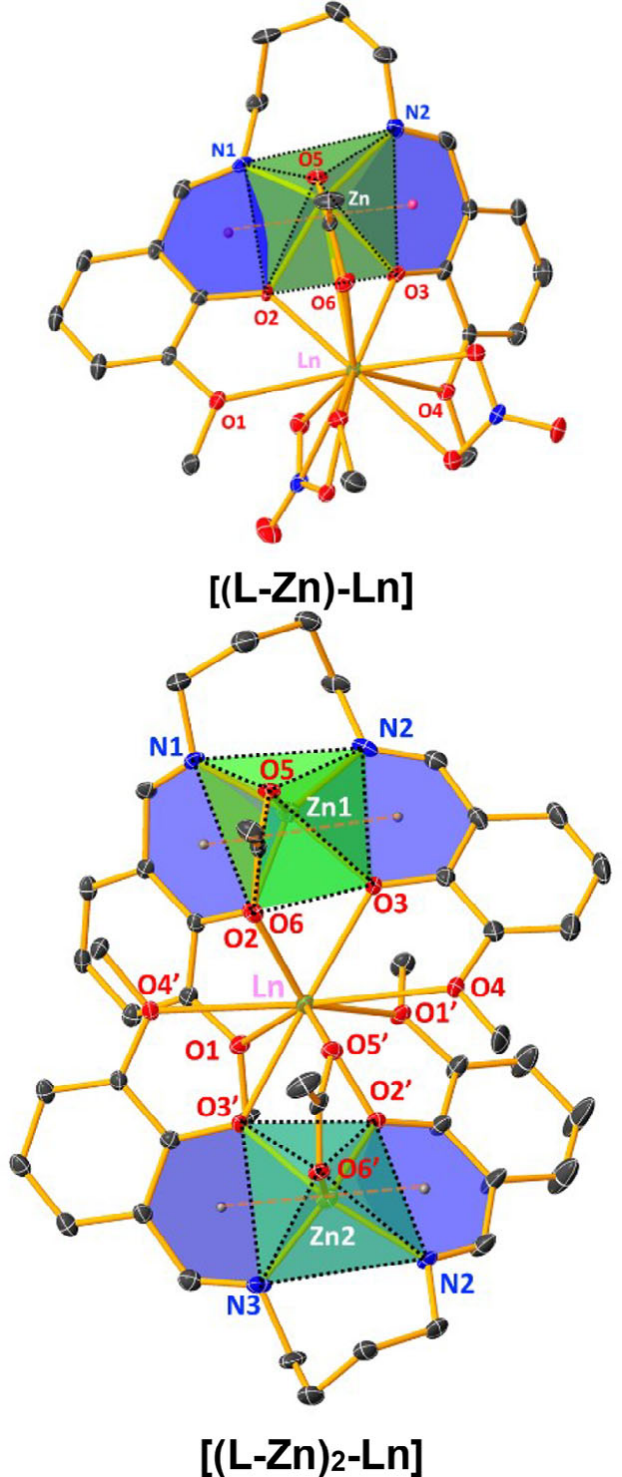
N1‐M‐N2	96.138	96.005	96.73	96.454	96.230	96.43	96.046	96.007	96.86	
N1‐M‐O2	89.005	89.018	87.03	88.665	88.757	86.73	89.135	89.028	87.14	
N1‐M‐O3	164.998	165.455	88.33	164.909	165.418	88.03	165.108	165.540	87.55	
O2‐M‐O3	76.673	76.799	77.02	76.921	77.017	77.43	76.709	76.881	76.43	
O3‐Ln‐O4	58.940	57.638	59.95	58.687	57.393	59.46	59.064	57.708	60.53	
O4‐Ln‐O1	131.850	133.466	114.81	131.949	133.960	134.02	131.830	133.361	134.03	
O1‐Ln‐O2	59.343	59.390	59.64	59.355	59.251	59.32	59.118	59.374	59.93	
O2‐Ln‐O3	62.144	62.297	64.32	62.433	62.439	64.52	62.138	62.255	65.13	
**Distances (Å)**										
M‐N1	2.011	2.084	2.048	2.103	2.078	2.062	2.107	2.086	2.043	
M‐N2	2.049	2.049	2.057	2.045	2.050	2.071	2.047	2.059	2.068	
M‐O2	2.019	2.022	2.038	2.019	2.016	2.052	2.021	2.019	2.038	
M‐O3	2.097	2.080	2.075	2.089	2.073	2.076	2.073	2.077	2.078	
M‐O5	2.011	2.007	2.005	2.009	2.005	1.996	2.011	2.008	1.988	
Ln‐O1	2.696	2.676	2.743	2.696	2.670	2.740	2.701	2.681	2.725	
Ln‐O2	2.487	2.456	2.402	2.475	2.447	2.414	2.487	2.453	2.365	
Ln‐O3	2.460	2.471	2.408	2.455	2.465	2.422	2.464	2.473	2.367	
Ln‐O4	2.772	2.826	2.736	2.773	2.826	2.716	2.773	2.831	2.731	
Ln‐O6	2.437	—	2.417	2.432	—	2.409	2.440	—	2.361	
Ln‐O7	2.414	—	2.520	2.406	—	2.520	2.414	—	2.362	
Dihedral angle	27.461	31.340	21.562	27.394	31.258	22.015	27.417	31.524	21.023	
Plane angle	52.713	60.232	52.941	52.601	60.212	52.494	52.675	60.280	52.931	
Centroid	3.670	3.609	3.626	3.672	3.609	3.649	3.668	3.612	3.621

The bimetallic and trimetallic complexes exhibit a significant structural change in the binding units. In the case of the bimetallic complex, the crystallographically independent unit comprises a Zn(II) and Ln(III), specifically [Zn(***μ***‐**L**)(***μ***‐CH₃COO)SLn‐(NO₃)₂], where **L** stands for ligand. The Ln element is Pr in **[(L‐Zn)‐Pr]**, Nd in **[(L‐Zn)‐Nd]**, and Sm in **[(L‐Zn)‐Sm]**, with S representing solvent, CH₃OH. Conversely, in the trimetallic complexes, the crystallographically independent unit consists of two Zn(II) and one Ln(III), specifically [Zn₂(***μ***‐L)₂(***μ***‐CH₃COO)₂Ln], with the Ln element being Pr in **[(L‐Zn)_2_‐Pr]**, Nd in **[(L‐Zn)_2_‐Nd]**, and Sm in **[(L‐Zn)_2_‐Sm]**. Structural data suggests that the counter anion (nitrate group and methanol) in the bimetallic complexes was replaced by an additional Zn‐L (antenna) unit in the trimetallic complexes. This may reduce deactivation through the nonradiative relaxation process. However, the presence of a free nitrate group in the trimetallic complex enhances its stability by contributing a negative charge to the charge neutralization process.

### 3.3 Crystal Packing Analysis

**Figure 2:**
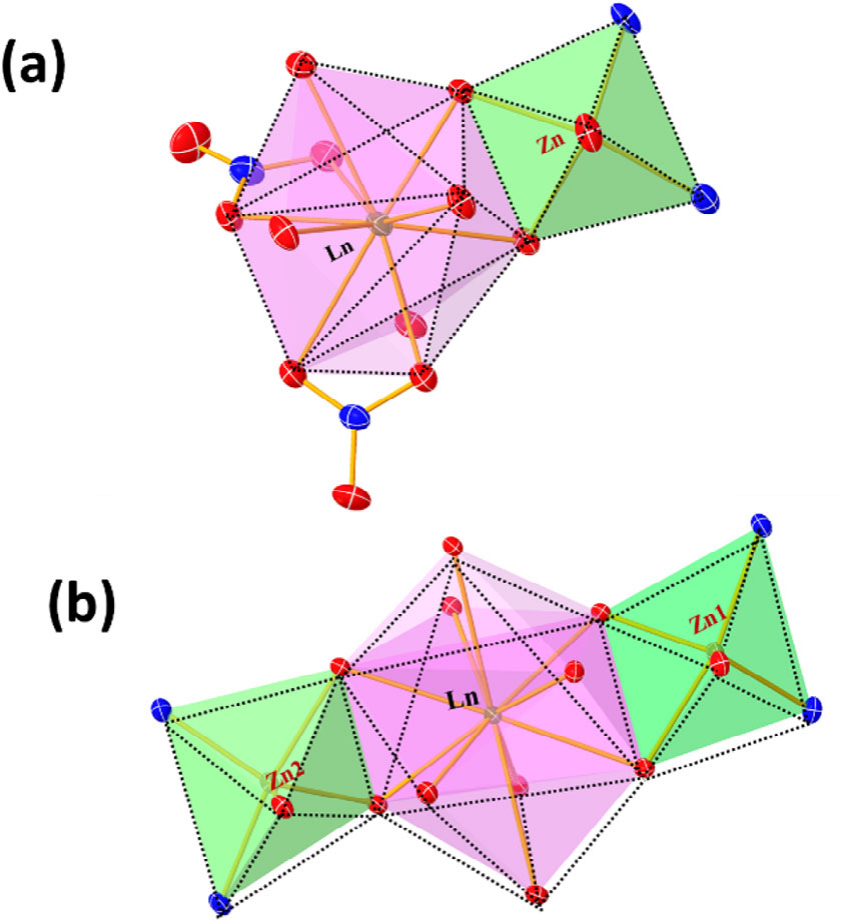
Polyhedron diagram shows the coordination environment (a) **[(L‐Zn)‐Ln]** and (b) **[(L‐Zn)_2_**‐**Ln]**, Ln=Pr, Nd, and Sm. Bicapped square antiprismatic for Ln(III) and distorted square pyramidal geometry for Zn (II) have been observed.

Both bimetallic and trimetallic complexes exhibit similar structural characteristics, categorized into two groups based on their coordination environments. The complexes [(**L‐Zn)‐Ln**] and [(**L‐Zn)₂‐Ln**] share analogous coordination geometries, with key differences in the coordinating ligands, particularly around the lanthanide center. In both complexes, the Zn(II) ions exhibit a distorted square planar geometry, as depicted in Figure 2. The square planar positions are occupied by two phenoxo oxygen atoms (O_2_ and O_3_ for [(**L‐Zn)‐Ln**], O_2_, O_3_ for Zn_1_, and O_6_, O_7_ for Zn_2_ in [(**L‐Zn)₂‐Ln**]), as well as two nitrogen atoms (N_1_, N_2_ for [(**L‐Zn)‐Ln**], N_1_, N_2_ for Zn_1_, and N_3_, N_4_ for Zn_2_ in [(**L‐Zn)₂‐Ln**]). The pyramidal position is occupied by a bridging acetate oxygen atom (O_11_ for [(**L‐Zn)‐Ln**], O_9_ for Zn_1_, O_11_ for Zn_2_ in [(**L‐Zn)₂‐Ln**]).

On the other hand, the Ln(III) ion adopts a bicapped square antiprismatic geometry with ten coordinated oxygen atoms. The ligands coordinating the Ln(III) ion differ between the two complexes. In the bimetallic **[(L‐Zn)‐Ln**] complex, four oxygen atoms originate from the ligand: two phenoxo oxygen atoms (O_2_ and O_3_) and two methoxy oxygen atoms (O_1_ and O_4_). Additionally, four oxygen atoms are contributed by two bidentate nitrate groups (O_5_, O_6_, O_8_, and O_9_), one oxygen atom from a bridging acetate group (O_12_), and the final oxygen from a methanol solvent molecule (O_13_).

In contrast, the trimetallic **[(L‐Zn)₂‐Ln**] complex has all eight coordinating oxygen atoms derived from the ligand, replacing the nitrate and methanol groups found in the bimetallic complex. In **[(L‐Zn)₂‐Ln**], the Ln(III) ion is coordinated by eight oxygen atoms from the ligand: four from Zn_1_, including two phenoxo oxygen atoms (O_2_ and O_3_) and two methoxy oxygen atoms (O_1_ and O_4_), and four from Zn_2_, including two phenoxo oxygen atoms (O_6_ and O_7_) and two methoxy oxygen atoms (O_5_ and O_8_). The remaining two oxygen atoms are contributed by the bridging acetate group (O_10_ and O_11_). This configuration leads to a more extensive coordination environment around the lanthanide ion in the trimetallic complexes.

**Figure 3:**
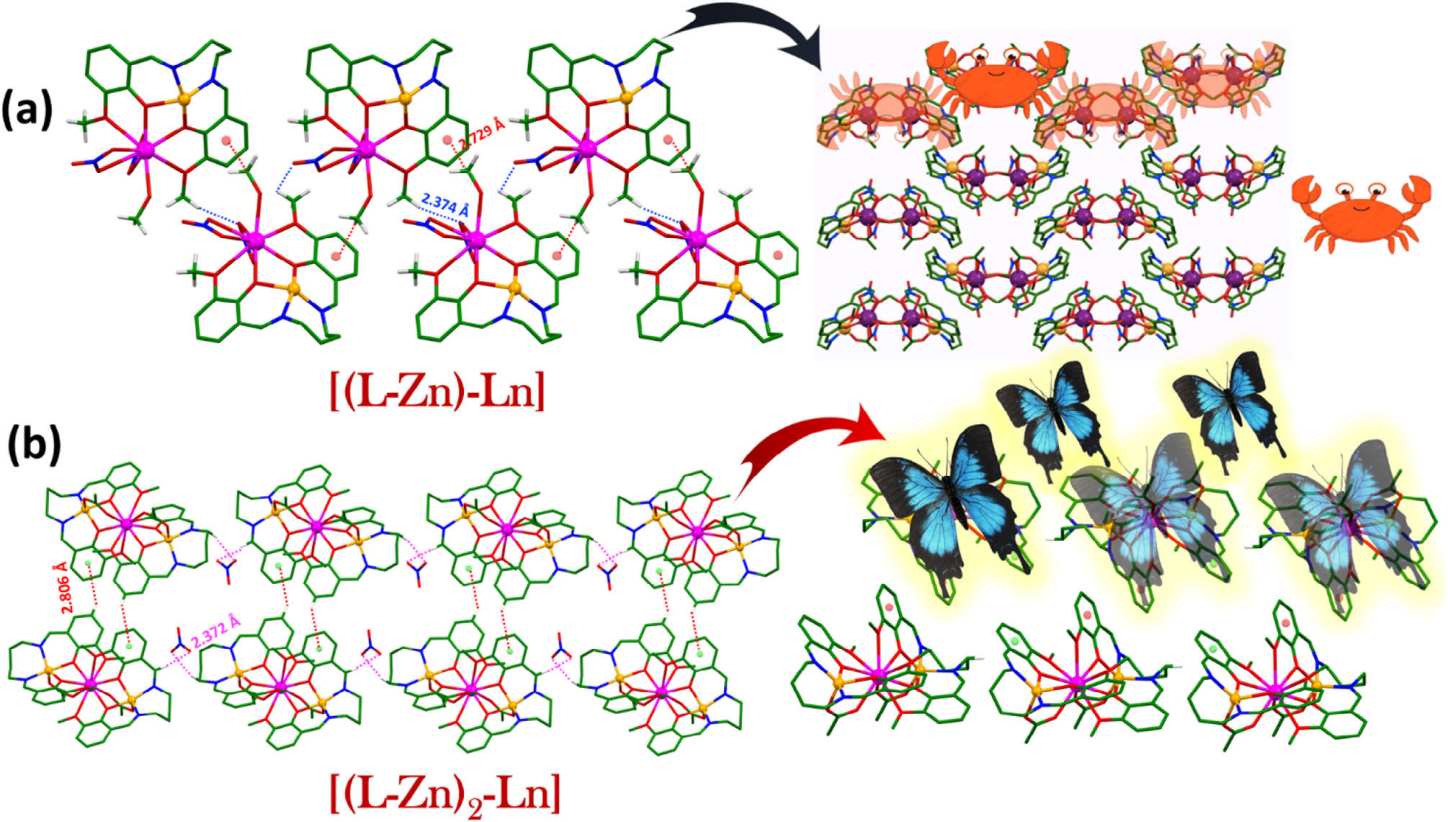
Important interaction and packing diagram for (a) **[(L‐Zn)‐Ln]** showing C─H···π and two C─H···O interactions (showing crab type of packing) and (b) **[(L‐Zn)_2_‐Ln]** showing C─H···π and C─H···O interactions (showing butterfly type of packing).

Most of the interactions present in both **[(L‐Zn)‐Ln**] and [(**L‐Zn)₂‐Ln**] complexes, such as C─H···π and C─H···O interactions, are shared across both derivatives. However, the propagation of these interactions differs significantly between the two complexes. As depicted in Figure S5 and Figure 3, the bimetallic complexes primarily exhibit two key interactions: (**1**) a C─H···π interaction (2.729 Å) between the methanolic CH₃ group and the salicylaldehyde ring, and (**2**) a C─H···O interaction (2.374 Å) between the hydrogen of the methoxy (OCH₃) group and the oxygen atom of the nitrate group, as illustrated in Figure 3a.

On the other hand, the trimetallic complexes exhibit two prominent interactions: (**1**) a C─H···π interaction (3.214 Å) between the sp^3^ CH₂ group (amine moiety, C10) and the salicylaldehyde ring, which facilitates the formation of a 1D chain, and (**2**) a π···π interaction (3.601 Å) between two salicylaldehyde rings, which leads to 2D packing (shown in Supporting Information Figure S5). Additionally, these complexes exhibit further stabilization through additional interactions where the free nitrate ions acting as a bridging between the two complex moieties, as illustrated in Figure 3b: (**1**) a (C─H···O‐NO‐O···H─C) interaction (2.372 Å) between the sp^3^ CH₂ group (amine moiety, C9), both the oxygen atom of the nitrate ion (present as a counter anion) and the CH (C8) present in imine bond propagating a 1D chain, and (**2**) a C─H···π interaction (2.806 Å) between the sp^2^ CH group of one salicylaldehyde ring and another salicylaldehyde ring, promoting 2D packing.

These interactions provide comparatively more stabilization than those observed in the bimetallic complexes. This distinction in packing interactions significantly influences the solid‐state emission properties of the two types of complexes. In addition to these primary interactions, several weaker interactions are also present in the crystal packing, which are common across both derivatives.

### 3.4 Ultraviolet Photoelectron Spectroscopy (UPS) and X‐Ray Photoelectron Spectroscopy (XPS) Studies

**Figure 4:**
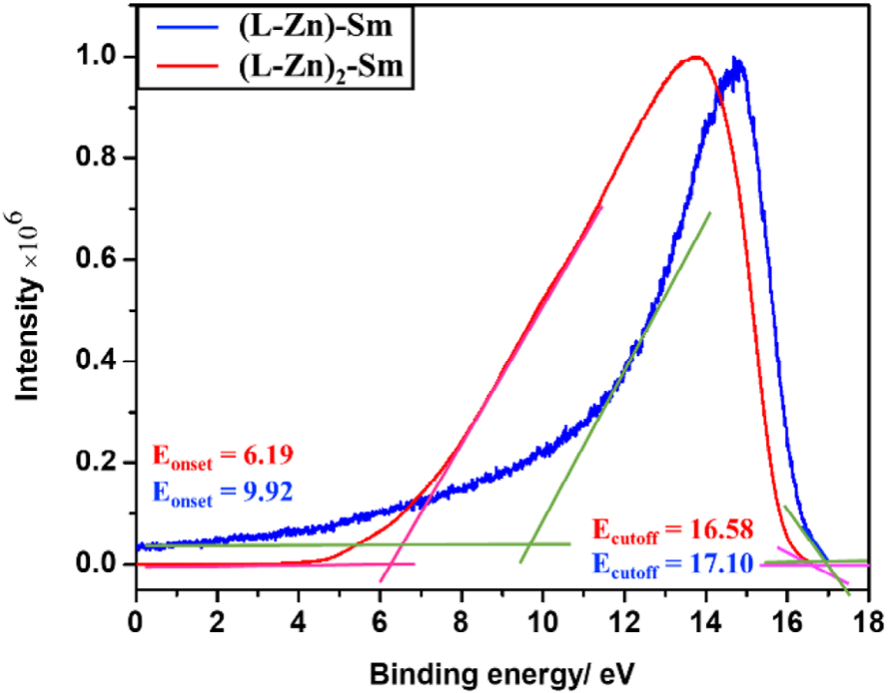
UPS spectra analysis for valence‐band regions (*E*_onset_) for **[L‐Zn‐Sm]** (blue) and **[(L‐Zn)_2_‐Sm]** (red) complexes.

UPS measurements were employed to determine the energy‐level alignment between the two complexes **[(L‐Zn)‐Sm]** and **[(L‐Zn)_2_‐Sm]**. The UPS spectra for the valence‐band regions are displayed in Figure 4. The **[(L‐Zn)‐Sm]** complex exhibited an *E*_onset_ of 9.92 eV, while the **[(L‐Zn)_2_‐Sm]** complex showed an *E*_onset_ of 6.19 eV. Additionally, the *E*_cutoff_ for **[(L‐Zn)‐Sm]** was found to be 17.10 eV, compared to 16.58 eV for **[(L‐Zn)_2_‐Sm]**. These values allow for the calculation of the valence band maxima (VBM) using the provided equation:

$$\mathrm{VBM}=h\nu -\left(E_{\mathrm{cutoff}}-E_{\mathrm{onset}}\right)$$

where *hν *= 21.22 eV is the incident photo energy for a He (I) source of UOS measurement systems.

The VBMs for the two complexes are 14.04 eV and 10.83 eV for **[(L‐Zn)‐Sm]** and **[(L‐Zn)_2_‐Sm]**, respectively.

The sample compositions align with the results obtained from the XPS semiquantitative analysis of the surface layers. The XPS survey spectra for the bimetallic complex **[(L‐Zn)‐Sm]** and trimetallic complex **[(L‐Zn)_2_‐Sm]** are presented in Supporting Information Figure S6, showing all characteristic electronic transitions of Sm, Zn, N, O, and C. The binding energy values for each individual element are listed in Supporting Information Table S1. At higher binding energies, the splitting of Ln‐3d peaks is observed due to the coupling of 3d and 4f hole states. Furthermore, it is realized that Sm‐3d has the presence of a doublet line, Sm 3d^3/2^ and Sm 3d^5/2^ peaks at 1081 and 1108 eV, as shown in Supporting Information Figure S6. The binding energy of Ln 3d or 4d electrons is closely related to the central atom's charge. Hence, its valency, determining the valency based solely on this criterion, is challenging because the intervals for different oxidation states may overlap. Zn(II) exhibits two bands corresponding to Zn 2p^3/2^ and Zn 2p^1/2^, respectively, observed in both complexes.

Oxygen, despite having three different sources namely, the nitrate group, the acetate group, only one similar chemical environment is observed in the complexes. The carbon peaks show a broadband (sp^2^ C) consisting of a shoulder peak (C─O) and two sharp peaks (π → π and π → π*) with relative intensities in the ratio 1:2:3, as indicated by the full width at half maximum (FWHM) values in Table S1.

**Figure 5:**
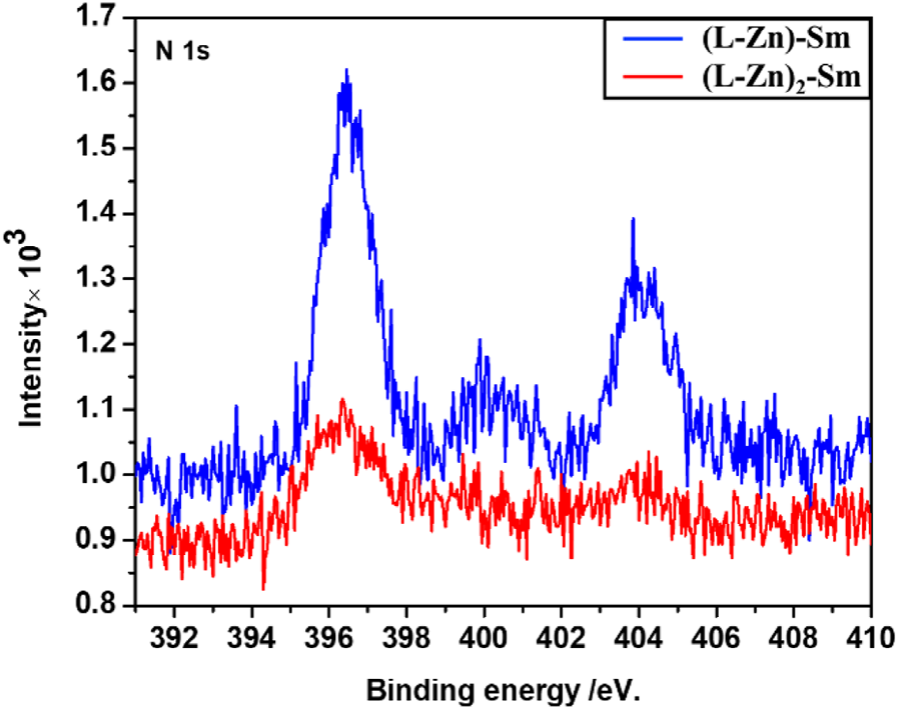
X‐ray photoelectron spectroscopy N 1s spectrum of the complexes. **[(L‐Zn)‐Sm]** and **[(L‐Zn)_2_‐Sm]** are presented.

For **[(L‐Zn)‐Sm]**, three bands of nitrogen were observed around 396, 400, and 404 eV, which correspond to N 1s (free), N 1s (attached with metal ion as nitrate), and N 1s (free nitrate ion).^[^ ^48^ ^]^ But in the case of **[(L‐Zn)_2_‐Sm]** the band ∼400 eV was not observed due to the absence on any coordinated nitrate group with the central lanthanide ion (shown in Figure 5). It further confirms the structure of trimetallic newly synthesized complexes.

There is clear evidence that the introduction of another antenna (Zn‐L) severely affects the valence characteristic of the complex and can significantly influence its optical properties, which in turn could have a substantial impact on the performance of devices such as OLEDs. Consequently,^[^ ^42^ ^]^ it is crucial to conduct detailed photophysical studies to understand these effects fully.

### 3.5 Thermal Studies

#### 3.5.1 TGA and DSC Studies

**Figure 6:**
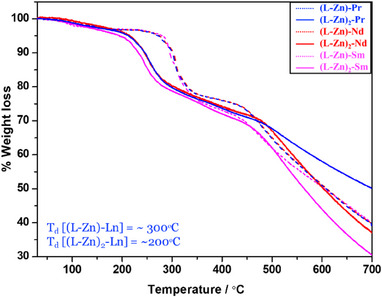
TGA curves of binuclear and trinuclear complexes.

TGA studies were conducted to evaluate the thermal stability of the bimetallic and trimetallic lanthanide complexes using TG‐DSC analysis. The TGA provided insight into the thermal decomposition behavior of the Ln complexes.^[^ ^49^ ^]^ As shown in Figure 6, the thermogram illustrates the heating behavior of the lanthanide complexes over a temperature range of 25–400 °C, revealing stages of solvent release and ligand decomposition. A thermal event between 65–150 °C corresponds to the release of water molecules from the lanthanide coordination network, accounting for approximately 30% mass loss, which is consistent with the elimination of two water molecules per complex, as depicted in Figure 6. Following this, in the 150–200 °C range, the trimetallic complexes undergo a second decomposition event, attributed to the release of the bridged acetate ligands and the butane chain within the ligand (**L**) framework, resulting in an additional 20% mass loss. This mass reduction corresponds to losing two acetate ligands and two butane chains from the ligand (**L**) structure (Figure 6).

**Figure 7:**
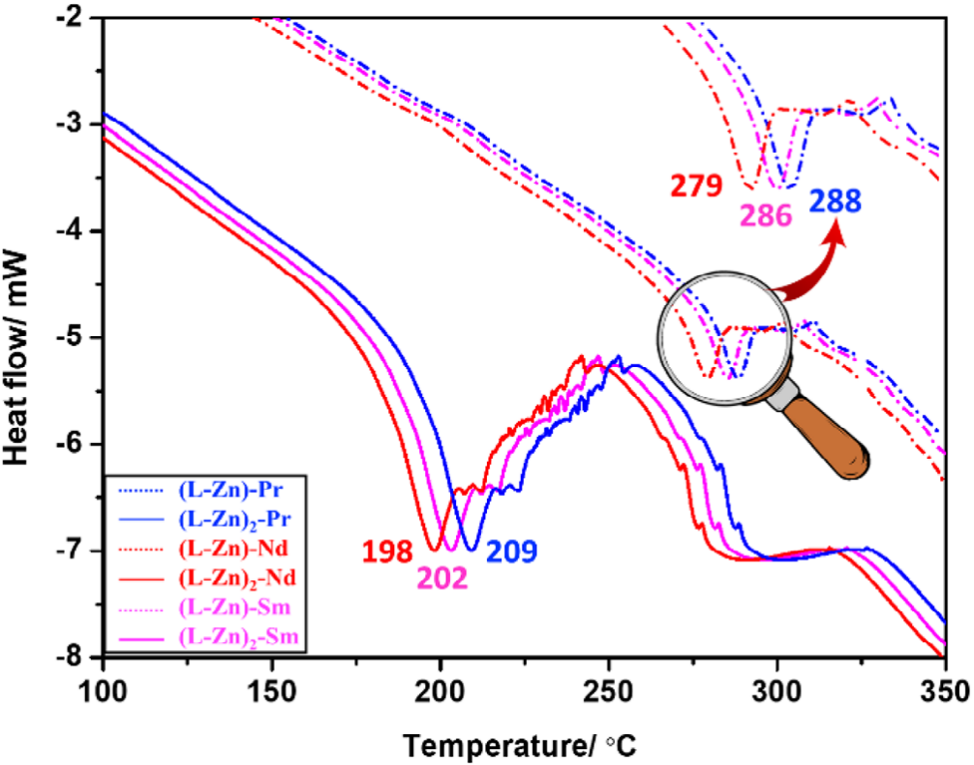
DSC curves of binuclear (dotted lines) and trinuclear (solid lines) complexes.

For the bimetallic complexes, decomposition begins around 300 °C, with a 20% mass loss due to the release of nitrate and methanol, which serve as coordinating ligands in the **[(L‐Zn)‐Ln**] complexes. Afterward, decomposition continues gradually, with the remaining complexes leaving a final residue of 30–50% by mass (shown in Figure 6).

The DSC analysis was done to study the phase change in both bimetallic and trimetallic complexes. In the case of trimetallic complexes, the DTA curve shows two endothermic peaks. The first thermal effect with *T_max_* = 209, 198, and 202 °C is due to the melting of the **[(L‐Zn)_2_‐Pr]**, **[(L‐Zn)_2_‐Nd]**, and **[(L‐Zn)_2_‐Sm]**, respectively. The endothermic peak with *T_max_* = ∼290 °C corresponds to the volatilization of the complexes.^[^ ^49^ ^]^ In the case of bimetallic complexes, the DSC profile shows a narrow endothermic peak with a *T_max_* value of 288, 279, and 286 °C for **[(L‐Zn)‐Pr]**, **[(L‐Zn)‐Nd]**, and **[(L‐Zn)‐Sm]**, respectively This probably corresponds to the melting and volatilization of the complexes (shown in Figure 7.

### 3.6 Photophysical Studies

#### 3.6.1 UV–vis Absorption Spectroscopic Studies

**Figure 8:**
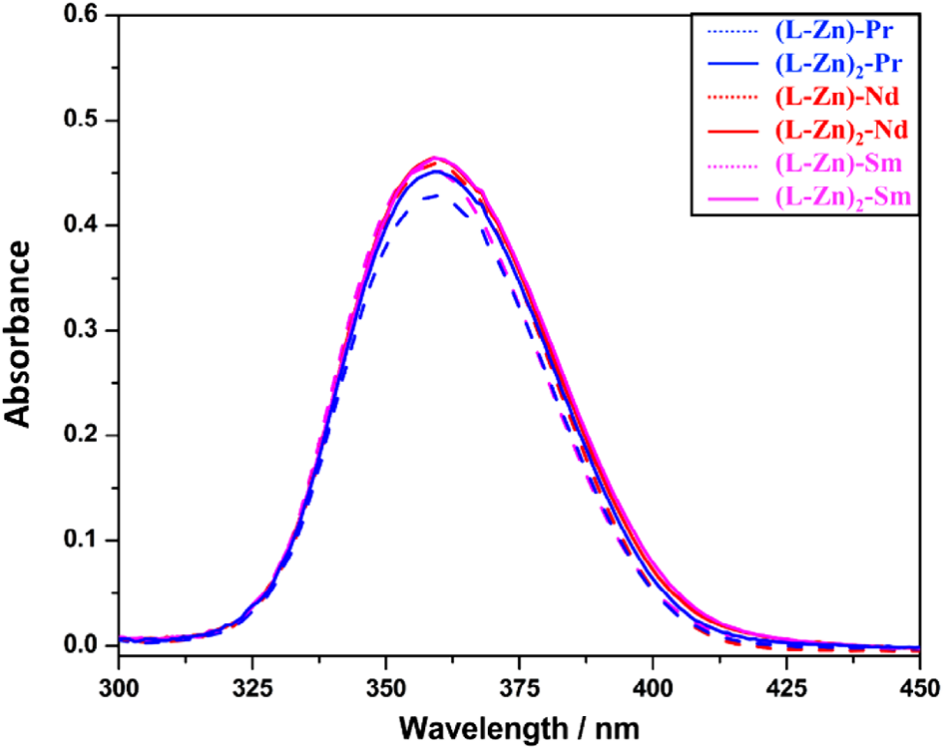
Absorption spectra for bimetallic (dotted lines) and trimetallic (solid lines) complexes.

To investigate the absorbance spectra of all complexes, each complex was dissolved in methanol to achieve a concentration of ∼1 × 10^−5^ M. Our previous research indicated that the free ligands displayed a prominent absorption band at 420 nm (n−π* transition) along with higher energy bands at 330, 292, and 260 nm (π–π* transitions), shown Supporting Information Figure S7.^[^ ^42^, ^43^, ^44^ ^]^ Upon complexation, all the bimetallic metal complexes exhibited a blue shift in their absorption spectra, with characteristic bands observed at 229 and 274 nm (intraligand charge transfer), as well as 360 nm due to ligand‐to‐metal charge transfer (LMCT) (shown in Supporting Information Figure S6). This blue shift aligns with previous findings,^[^ ^50^, ^51^, ^52^ ^]^ although some studies have noted red shifts under different conditions.^[^ ^47^ ^]^ A similar trend was observed in the trimetallic complexes, with all metal complexes maintaining their characteristic absorbance band around 360 nm, indicating that the absorbance band was not influenced by an increase in the antenna effect (as illustrated in Figure 8). The observation that all metal complexes, including the trimetallic complexes, maintain a characteristic absorbance band around 360 nm is significant. This stability suggests that the fundamental electronic transitions responsible for this band remain unaffected by the presence of additional metal centers, indicating a stable overall electronic structure and preserving the specific LMCT transition.^[^ ^53^ ^]^

#### 3.6.2 Steady‐State Luminescence Studies

**Figure 9:**
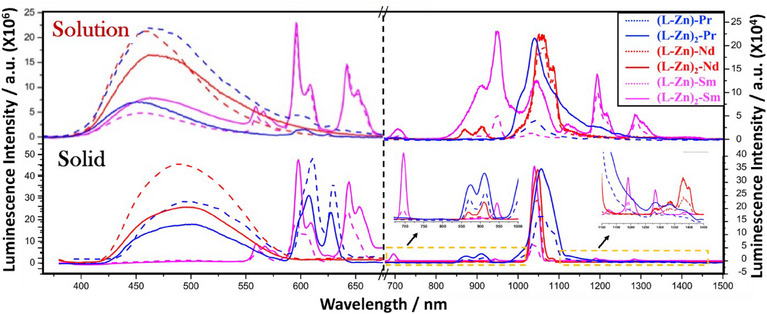
Luminescence spectra of lanthanides complexes in (a) methanolic solution and (b) solid state. Spectra having solid lines represents the trimetallic complexes, while the broken line represents the bimetallic complexes. The electronic energy levels involved in each f → f transition in lanthanide complexes are also mentioned in Supporting Information Table S2. The excitation wavelength was 360 nm in each case. Optical density at excitation wavelength was kept at about 0.45 for all complexes.

The luminescence properties of the complexes were studied in both the solution (∼1 × 10^−5^ M, methanol) and the solid state, as depicted in Figure 9. In solution on excitation at 360 nm, all complexes show emission around ∼460 nm attributed to (LMCT), which shifts to ∼490 nm in the solid state. This red shift in LMCT spectra in the solid state is likely due to multiple packing interactions (C─H···π, C─H···O, and π–π) that alter the electronic environment, shifting emission to longer wavelengths. Notably, the bimetallic complexes exhibit luminescence intensities that are 2 to 3 times higher than those of the trimetallic complexes. However, the reverse trend is observed in f–f transitions, where the trimetallic complexes tend to have stronger emissions intensity than the bimetallic complexes.

Both the bimetallic [**(L‐Zn)‐Pr**] and trimetallic [**(L‐Zn)₂‐Pr**] praseodymium complexes display six characteristic bands: ^1^D₂→^3^H₄ (*λ*_em_ = 600 nm); ^3^P₀→^3^H₆ (*λ*_em_ = 623 nm); ¹D₂→^3^F₄ (*λ*_em_ = 1028 nm); ¹G₄→¹H₄ (*λ*_em_ = 1338 nm); ¹G₄→¹H₅ (*λ*_em_ = 1372 nm); ¹G₄→¹H₆ (*λ*_em_ = 1407 nm). Similarly, the neodymium complexes [**(L‐Zn)‐Nd**] and [**(L‐Zn)₂‐Nd**] exhibit three characteristic bands: ⁴F_3/2_→⁴I_9/2_ (*λ*_em_ = 870; 904 nm); ⁴F_3/2_→⁴I_11/2_ (*λ*_em_ = 1057 nm) and ⁴F_3/2_→⁴I_13/2_ (*λ*_em_ = 1333 nm). The samarium complexes [**(L‐Zn)‐Sm**] and [**(L‐Zn)₂‐Sm**] show seven f–f transition bands: ⁴G_5/2_→^6^H_5/2_ (*λ*_em_ = 559 nm); ⁴G_5/2_→^6^H_7/2_ (*λ*_em_ = 595 nm); ⁴G_5/2_→^6^H_9/2_ (*λ*_em_ = 645 nm); ⁴G_5/2_→^6^F_1/2_ (*λ*_em_ = 889 nm); ⁴G_5/2_→^6^F_5/2_ (*λ*_em_ = 942 nm); ⁴G_5/2_→^6^F_7/2_ (*λ*_em_ = 1021 nm); ⁴G_5/2_→^6^F_9/2_ (*λ*_em_ = 1161 nm).

Although no significant shifts in band positions are observed between the bimetallic (**[L‐Zn‐Ln]**) and trimetallic [**(L‐Zn)₂‐Ln**] complexes, their luminescence intensities are markedly influenced by the introduction of an additional antenna. As highlighted in Figure 9 and elaborated in Supporting Information Table S2, the luminescence intensities of the trimetallic complexes are significantly enhanced, particularly in the NIR region.

**Figure 10:**
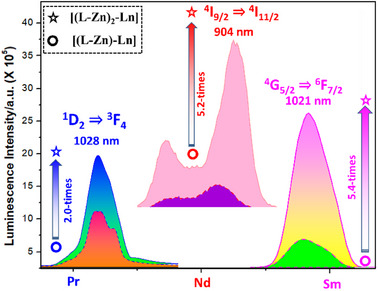
Shows the enhancement in NIR luminescence intensity with the addition of an extra antenna in the trimetallic complexes (in methanolic solution). ODs at excitation wavelength were kept the same for all the complexes).

In the case of the trimetallic complex [**(L‐Zn)₂‐Pr**], all transitions in the solution phase exhibited a 1‐ to 2‐times increase in intensity. The most significant enhancement was observed for the ¹D₂→^3^F₄ (*λ*_em_ = 1028 nm) transition, which showed a 2‐times increase in the NIR region (Figure 10 and Supporting Information Table S2).

For the neodymium complex [**(L‐Zn)₂‐Nd**], a 1.5‐ to 5‐times increase in f–f transition intensity was noted, with the maximum 5.2‐times increase observed for the ⁴F_3/2_→⁴I_9/2_ (*λ*_em_ = 870; 904 nm) transition in solution (refer to Supporting Information Table S2 and Figure 10). Similarly, the samarium complex [**(L‐Zn)₂‐Sm**] demonstrated a 1.5‐fold increase in intensity in the visible region (560–645 nm), while in the NIR region, the intensity increased by 2 to 5 times, with the highest enhancement of 5.4 times occurring for the ⁴G_5/2_→^6^F_7/2_ (*λ*_em_ = 1021 nm) transition.This indicates that the presence of an extra antenna notably boosts energy transfer, thereby improving overall emission efficiency.

The remarkable enhancement in luminescence observed in the trimetallic complexes is primarily due to the incorporation of an additional antenna, which plays a pivotal role in optimizing energy transfer to the lanthanide energy levels. This energy is subsequently transferred with greater efficiency to the lanthanide centers. Moreover, the reduction in energy dissipation through LMCT pathways (Figure 9) ensures that a larger portion of the absorbed energy is conserved and directed toward the lanthanide energy manifolds. This data was also further supported by theoretical evidence. The computed triplet state energy (T1) of the ligand (**L**) is 2.92 eV at the B3LYP/6‐31G(d, p) level, indicating a high‐lying triplet state (shown in Supporting Information Figure S8). Given that Ln(III) complexes typically exhibit multiple excited states in the 2.5–3.0 eV range, the ligand's T1 state is well‐positioned to facilitate energy transfer to the Ln(III) center, making it suitable for the sensitization process.^[^ ^54^ ^]^

This efficient energy funneling enhances the population of the lanthanide excited states, thereby boosting the overall emission intensity, particularly in the near‐infrared regions. The dual‐antenna system in trimetallic complexes also promotes synergistic interactions, further improving the energy transfer process and reducing competing nonradiative losses.

**Figure 11:**
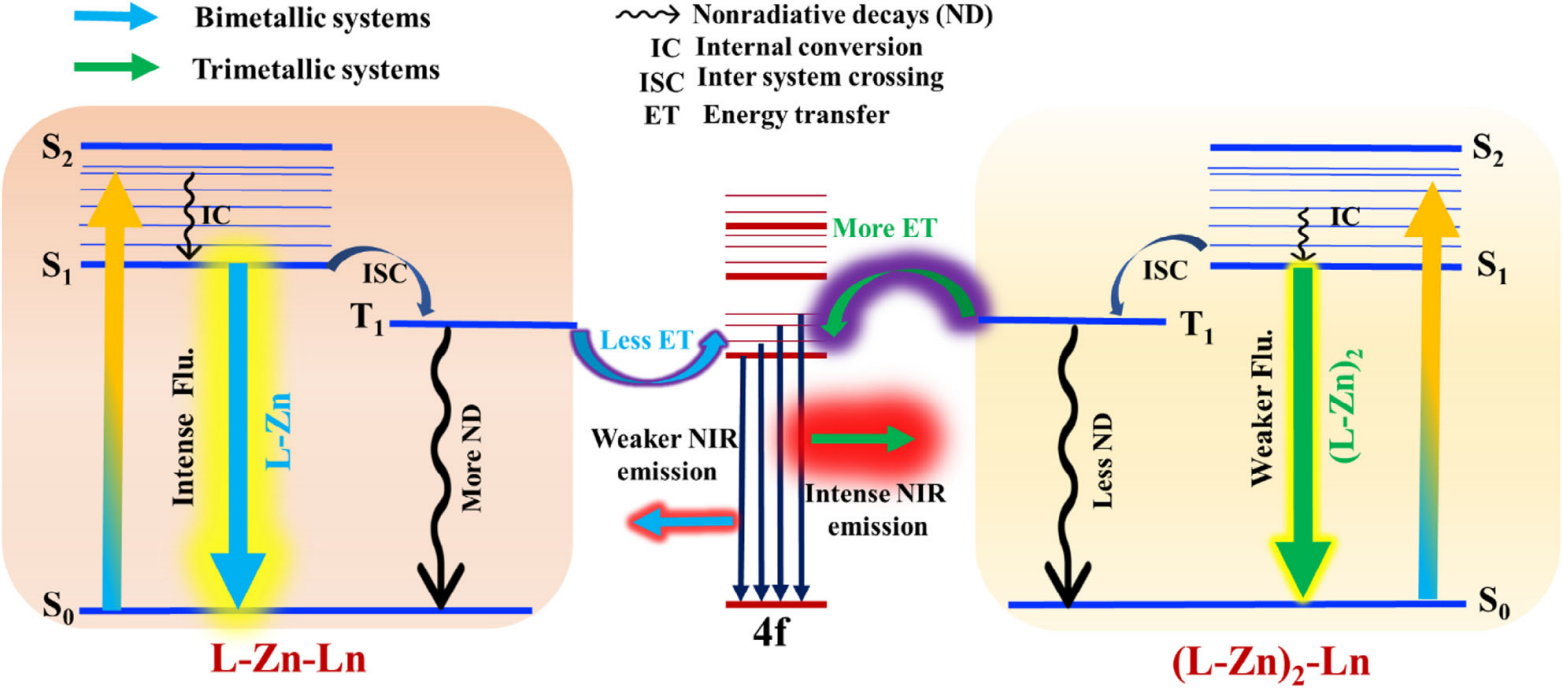
Pictorial diagram of the energy transfer process in bimetallic and trimetallic complexes.

These findings underline the critical role of complex architecture in tailoring luminescent properties, making such trimetallic systems highly promising candidates for applications in advanced photonic devices, such as near‐infrared‐emitting OLEDs or bioimaging agents. A pictorial diagram illustrating these energy transfer processes, including the roles of the antennas and lanthanide energy levels, is presented in Figure 11.

In both solution and solid states, a consistent trend of increasing emission intensity from the visible to the NIR region highlights the antenna's effective role in energy transfer to the lanthanide energy levels, regardless of the medium. In the visible region, trimetallic complexes show reduced luminescence compared to their bimetallic counterparts, likely due to energy redistribution. The additional antenna channels more energy toward the NIR region, resulting in diminished visible emission. In contrast, the trimetallic complexes exhibit significantly enhanced NIR emission, indicating that the antenna improves energy transfer efficiency to NIR‐active lanthanide levels. This suggests reduced energy loss through nonradiative pathways, allowing more energy to reach the lanthanide ions and thereby boosting NIR emission. The lack of peak shifts between the solution and solid states further confirms that the antenna's energy transfer mechanisms are consistent across both phases, continuing to capture excitation energy and funnel it effectively to the lanthanide energy manifolds. The antenna's ability to minimize energy losses and enhance luminescence efficiency, particularly in the NIR region, is evident across different lanthanide ions. This consistent enhancement in NIR emission, as shown in Figure 11 and Supporting Information Table S2, underscores the antenna's crucial role in improving energy transfer and reducing nonradiative losses in the trimetallic complexes.

**Figure 12:**
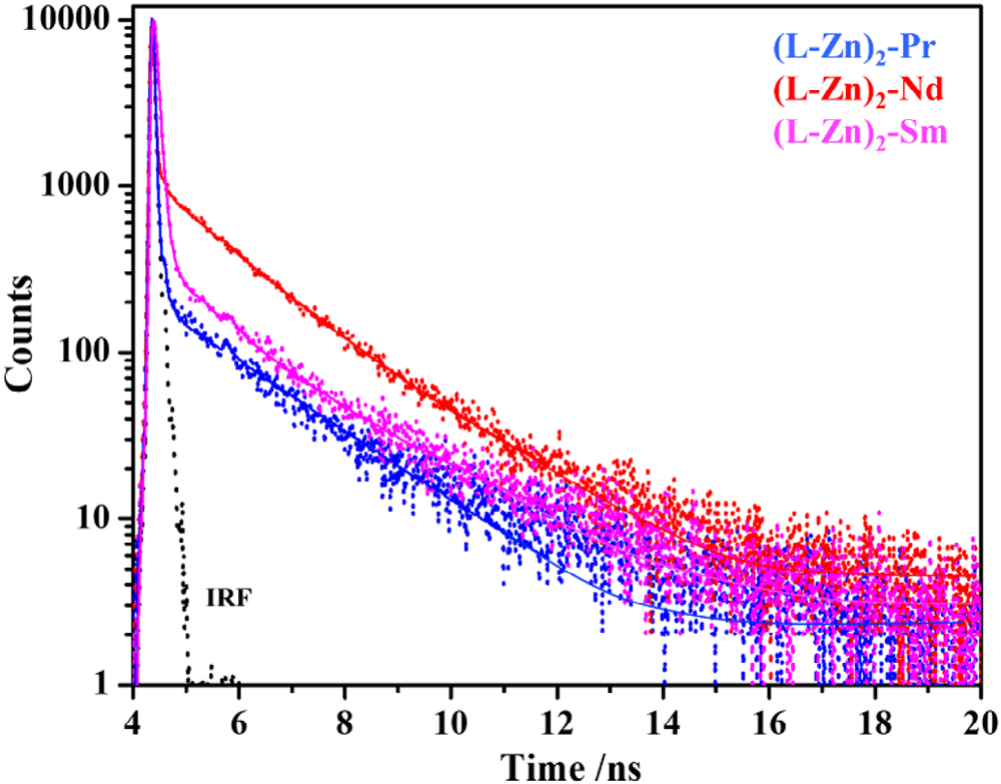
Luminescence decays of trimetallic lanthanide complexes in methanolic solution. The decays were obtained by exciting the solution with a 374 nm laser. The monitoring wavelength is 460 nm.

The time‐resolved photoluminescence (PL) decay analysis of bimetallic and trimetallic Ln complexes reveals a multi‐exponential decay behavior, indicating the presence of multiple excited‐state relaxation pathways. The luminescence decay profile in the solution is shown in Figure 12 and Supporting Information Figure S9, while the lifetime data are presented in Table 3. The three decay components (**τ**_1_, **τ**_2_, **τ**_3_) suggest a combination of radiative and nonradiative processes, with short‐lived components corresponding to fast depopulation mechanisms and longer‐lived components associated with radiative decay. The Pr (III) complexes, **[(L‐Zn)‐Pr]** and **[(L‐Zn)₂‐Pr]**, exhibit the shortest **τ**_**av**_ values (0.68 and 0.10 ns, respectively) and the lowest fluorescence quantum yields (φ_*F*_ = 0.79% and 0.28%), confirming that Pr (III) undergoes efficient nonradiative multiphonon relaxation, leading to rapid quenching. The Nd(III) complexes,**[(L‐Zn)‐Nd]** and **[(L‐Zn)₂‐Nd]**, show slightly longer **τ**_**av**_ values (0.26 and 0.85 ns) but still exhibit strong quenching, likely due to high‐energy vibrational coupling with solvent molecules, as supported by literature reports on Nd(III) luminescence.^[^ ^55^ ^]^ The Sm(III) complexes, **[(L‐Zn)‐Sm]** and **[(L‐Zn)₂‐Sm]**, display improved luminescence with longer **τ**_**av**_ values (0.38 and 0.67 ns, respectively) and higher φ_*F*_ values, particularly in **[(L‐Zn)₂‐Sm]** (1.96%), which benefits from reduced nonradiative losses and enhanced radiative transitions in the visible region. The trimetallic complexes **[(L‐Zn)₂‐Ln]** generally exhibit longer lifetimes and lower quantum yields compared to their bimetallic counterparts, suggesting that the increased rigidity of the coordination environment effectively suppresses vibrational quenching. This trend is well‐documented in previous studies, which emphasize that ligand rigidity and coordination geometry play a crucial role in enhancing lanthanide luminescence in the NIR region.^[^ ^56^ ^]^ Overall, these results confirm that transitioning from bimetallic to trimetallic complexes increases fluorescence lifetime, which limits strong nonradiative relaxation, making structural modifications essential for optimizing their optical applications.

**Table 3:** The luminescence lifetimes and quantum yield of lanthanide complexes in solution (methanol).

Compound	τ_1_/ns (A_1_)	τ_2_/ns (A_2_)	τ_3_/ns (A_3_)	τ_**av**_/ns	φ_*F*_ (%)
(L‐Zn)‐Pr	1.39 (22.44%)	4.09 (8.97%)	0.01 (68.58%)	0.68	0.79
(L‐Zn)‐Nd	1.37 (6.89%)	0.02 (89.55%)	4.27 (3.56%)	0.26	0.93
(L‐Zn)‐Sm	1.08 (8.68%)	0.051 (81.63%)	2.62 (9.68%)	0.38	1.40
(L‐Zn)_2_‐Pr	0.024 (40.02%)	0.02 (55.97%)	2.02 (4.01%)	0.10	0.28
(L‐Zn)_2_‐Nd	1.47 (33.67%)	3.76 (9.36%)	0.008 (57.03%)	0.85	0.88
(L‐Zn)_2_‐Sm	1.15 (13.33%)	3.21 (14.77%)	0.06 (71.91%)	0.67	1.96

## 4 Conclusion

The effect of introducing an additional light‐absorbing moiety (antenna) on the NIR luminescence properties of Lanthanide complexes has been elucidated by designing two sets of lanthanide complexes having an architecture of **[(L‐Zn)−Ln]**. Both sets are structurally fully characterized by SCXRD, PXRD, and XPS. Crystal packing analysis shows the presence of different packing for bimetallic and trimetallic lanthanide complexes due to substantially different interactions, which is supported by XPS studies. This difference in interaction also leads to quite different thermal properties. Steady‐state luminescence studies highlighted the substantial impact of the dual‐antenna system in the trimetallic complexes. While these systems exhibited reduced luminescence in the visible region compared to their bimetallic counterparts, a remarkable enhancement in NIR emission was observed. The enhanced NIR luminescence is attributed to the dual‐antenna mechanism, which improves energy transfer efficiency from the **Zn‐L** moieties to the lanthanide (**Ln**) excited states. This is also supported by the enhanced fluorescence lifetime upon transitioning from a bimetallic to a trimetallic system (especially in the case of Nd and Sm complexes), which suppresses nonradiative relaxation, emphasizing the role of ligand rigidity and coordination geometry. These findings provide valuable insights for optimizing luminescent materials for NIR applications. This enhancement positions the trimetallic complexes as promising candidates for applications in NIR‐emitting devices, such as OLEDs, where strong NIR emission is highly desirable.

## Conflict of Interests

The authors declare no conflict of interest.

## Supporting information



Supporting Information

## Data Availability

The data that support the findings of this study are available from the corresponding author upon reasonable request.
