# Supplementary material for: Dual‐Antenna Trimetallic Lanthanide Complexes for Enhanced Near‐Infrared Luminescence
Source: Chem Asian J. 2025 Jun 17;20(18):e00017. doi: 10.1002/asia.202500017 (PMC12450039; doi:10.1002/asia.202500017)
Supplement: Supplementary file 1 — Supporting Information [file ASIA-20-e00017-s001.docx]

**ESI**

**Dual-Antenna Trimetallic Lanthanide Complexes for Enhanced Near-Infrared Luminescence**

Krishanu Bandyopadhyay^1^, Abhineet Verma^2^, Satyen Saha^1^*

*^1^Department of Chemistry, Institute of Science, Banaras Hindu University, Varanasi-221005, India.*

*^2^Department of Chemistry, Malaviya National Institute of Technology (MNIT), Jaipur-302017, India^.^*

**Material and method**

3-Methoxysalicylaldehyde, 1,4-diaminobutane, Zn(CH_3_COO)_2_ 2H_2_O and Ln(NO_3_)_3_·6H_2_O (Ln = PrIII, NdIII, SmIII ) were purchased from Sigma-Aldrich and were used without any further purification. The solvents used in the synthesis and photophysical studies were dried following the standard procedure.^[S1]^ Infrared spectra were recorded on a PerkinElmer Spectrum-2 FTIR spectrometer using KBr pellets 400−4000 cm^−1^. The powder X-ray diffraction (PXRD) data were collected on a Bruker D8-ADVANCE diffractometer equipped with Cu Kα1 (λ = 1.5406 Å; 1600 W, 40 kV, 40 mA) at a scan speed of 5° min^−1^. Elemental analyses were measured on a Vario EL III elemental analyzer. For SCXRD data of **[(L-Zn)_2_ -Ln]** and **[(L-Zn) -Ln]** complexes were collected on a Rigaku Diffraction XtaLAB Synergy-i diffractometer using graphite-monochromatized Mo Kα radiation at 298 K (for **[(L-Zn)-Ln]**) and at 100 K (for **[(L-Zn)_2_ -Ln]**). The structures are solved by direct methods and refined by full-matrix least-squares on F^2^ using SHELX-2019. ^[S2]^ The non-hydrogen atoms are refined with anisotropic thermal parameters. All the hydrogen atoms are geometrically fixed and allowed to be refined using a riding model. Integrated intensity information for each reflection was obtained by reducing the data frames with the program APEX3.^[S3]^ The absorption correction program SADABS^[S4]^ was employed to correct the data for absorption effects. Drawings are made using OLEX-II^[S5]^ and MERCURY.^[S6]^ The X-ray photoelectron spectra (XPS) were obtained using ULVAC-PHI 5000 VeersaProbe III scanning XPS Microprobe. Indium-doped tin oxide (ITO) glass plate (1 × 3 cm) coated with composite ink. It is designed to scan a sample surface across 1.4 mm with a small spot X-ray beam of 10 to 200 μm. It also has ion cannons for depth profile analysis and UPS analysis (ultraviolet source, can produce He I or He II excitation). ^1^H NMR spectra were recorded on a JOEL Delta 500 MHz instrument, and chemical shifts are reported in parts per million (ppm) relative to a tetramethylsilane (TMS) standard. High-resolution mass spectrometry (HRMS) was recorded using a SCIEX X500R QTOF system powered by SCIEX OS software. Steady-state UV−visible absorption spectra were measured by Cary 100 Bio, Agilent, in the range of 200−800 nm. The instrument has photometric linearity until absorbance 3.5 and has a wavelength resolution of 0.2 nm. Fluorescence spectrophotometry (Fluorolog 3-21, Horiba Scientific) was used for fluorescence measurements in solution and solid states. The instrument is also equipped with a nitrogen-cooled UV−vis−NIR PMT detector (R5509−73, from Hamamatsu Photonics, Japan) for recording luminescence up to 1700 nm. For the measure of band intensity in the emission spectrum, the OD of the solution remains the same (0.45) for all the samples, and in the solid state, the data were repeated three times to check the repeatability of the data. The quantum yield of the samples was determined by comparing them with quinine sulfate (in 0.1 N H₂SO₄ solution) as a reference, ensuring the same optical density at the excitation wavelength as that of the samples. The time-correlated single photon counting (TCSPC) measurements were performed using a state-of-the-art TCSPC spectrometer (Horiba Scientific, DeltaFlex^TM^, modular fluorescence lifetime system) equipped with a 374 nm nanosecond pulse diode laser. The amplitude average lifetime ($\boldsymbol{\tau}_{\boldsymbol{avg}}$) of the excited state for complexes in MeOH has been calculated using the following equation:

$\boldsymbol{\tau}_{\boldsymbol{avg}}$ **=** $\frac{\sum_{\boldsymbol{i=1}}^{\boldsymbol{n}} \boldsymbol{A}_{\boldsymbol{i}}\boldsymbol{\tau}_{\boldsymbol{i}}}{\sum_{\boldsymbol{i=1}}^{\boldsymbol{n}} \boldsymbol{A}_{\boldsymbol{i}}}$ **,** where, $\sum_{\boldsymbol{i=1}}^{\boldsymbol{n}} \boldsymbol{A}_{\boldsymbol{i}}$ **= 1**

Here, 𝜏_i_ denotes the lifetimes of the i^th^ component, and A_i_ shows their corresponding percentage contribution with respect to decay time.

**Synthesis of [(L-Zn)_2_-Nd] and [(L-Zn)_2_-Sm]**

[(**L-Zn)_2_-Nd**]: 20 mL of a methanolic solution of **L** (0.71 g, 2.0 mmol) was added to 15 mL of a methanolic solution of Zn- (CH_3_COO)_2_·2H_2_O (0.44 g, 2.0 mmol) while stirring, followed by dropwise addition of a 15 mL methanolic solution of Nd(NO_3_)_3_·6H_2_O (0.44 g, 1.0 mmol). The reaction mixture was kept stirring for 3 hrs. The reaction mixture was then cooled to RT and filtered to eliminate any unreacted starting materials. The filtrate was kept for crystallization using a slow evaporation method under a controlled environment (having 15° to 20 °C temperature). Light yellow needle-shaped crystals for [**Nd**] suitable for single crystals for SCXRD measurements were obtained within a week.

[(**L-Zn)_2_-Sm**]: 20 mL of a methanolic solution of **L** (0.71 g, 2.0 mmol) was added to 15 mL of a methanolic solution of Zn- (CH_3_COO)_2_·2H_2_O (0.44 g, 2.0 mmol) while stirring, followed by dropwise addition of a 15 mL methanolic solution of Sm(NO_3_)_3_·6H_2_O (0.44 g, 1.0 mmol). The reaction mixture was kept stirring for 3 hrs. The reaction mixture was then cooled to RT and filtered to eliminate any unreacted starting materials. The filtrate was kept for crystallization using a slow evaporation method under a controlled environment (having 15° to 20 °C temperature). Light yellow needle-shaped crystals for [**Nd**] suitable for single crystals for SCXRD measurements were obtained within a week.

| 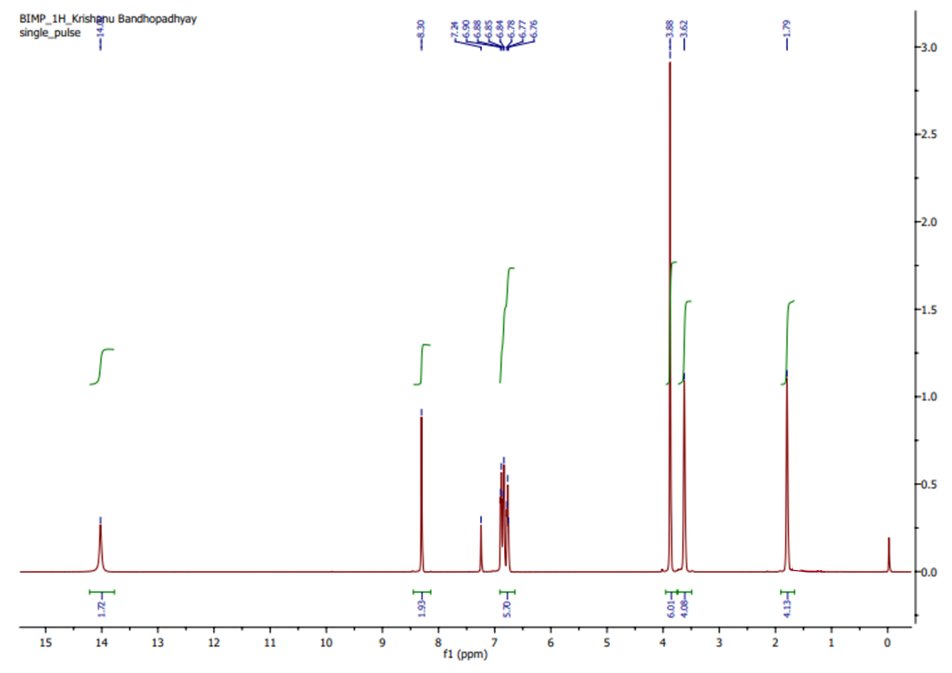  **Ligand** |
| --- |
| 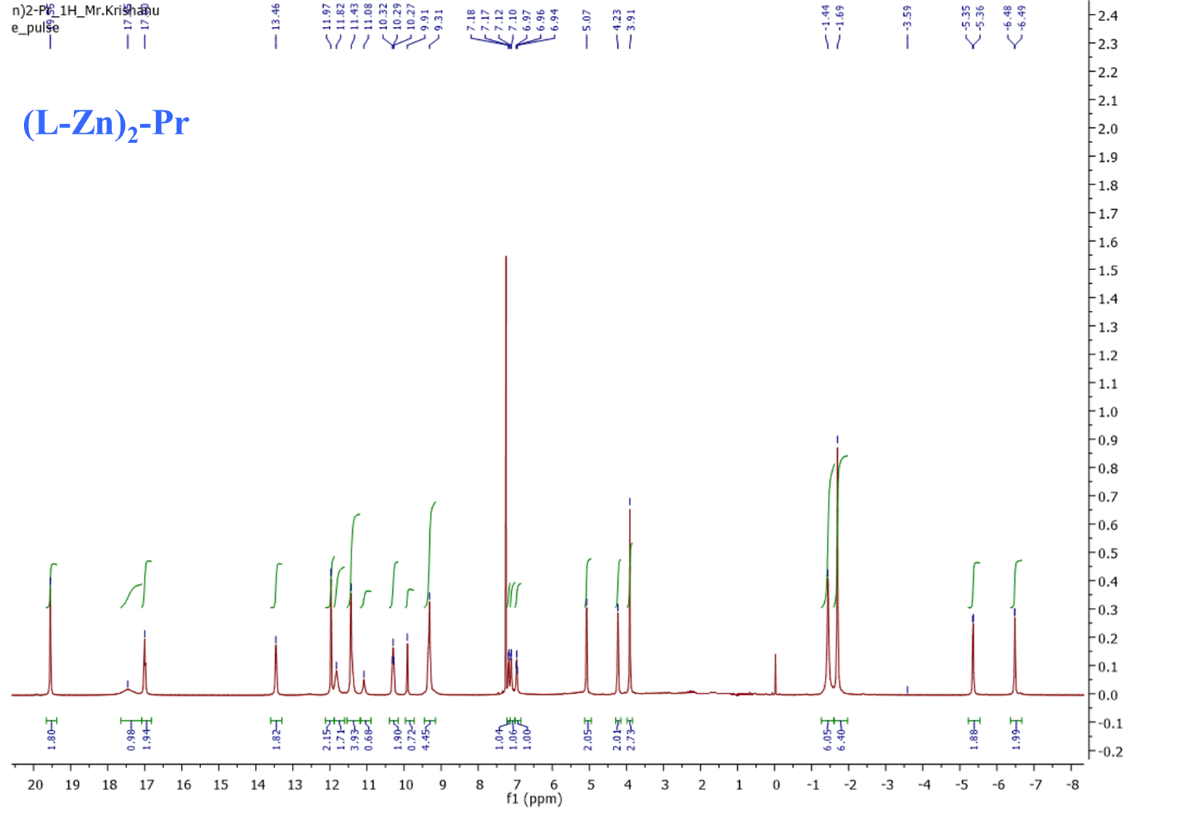 |
| 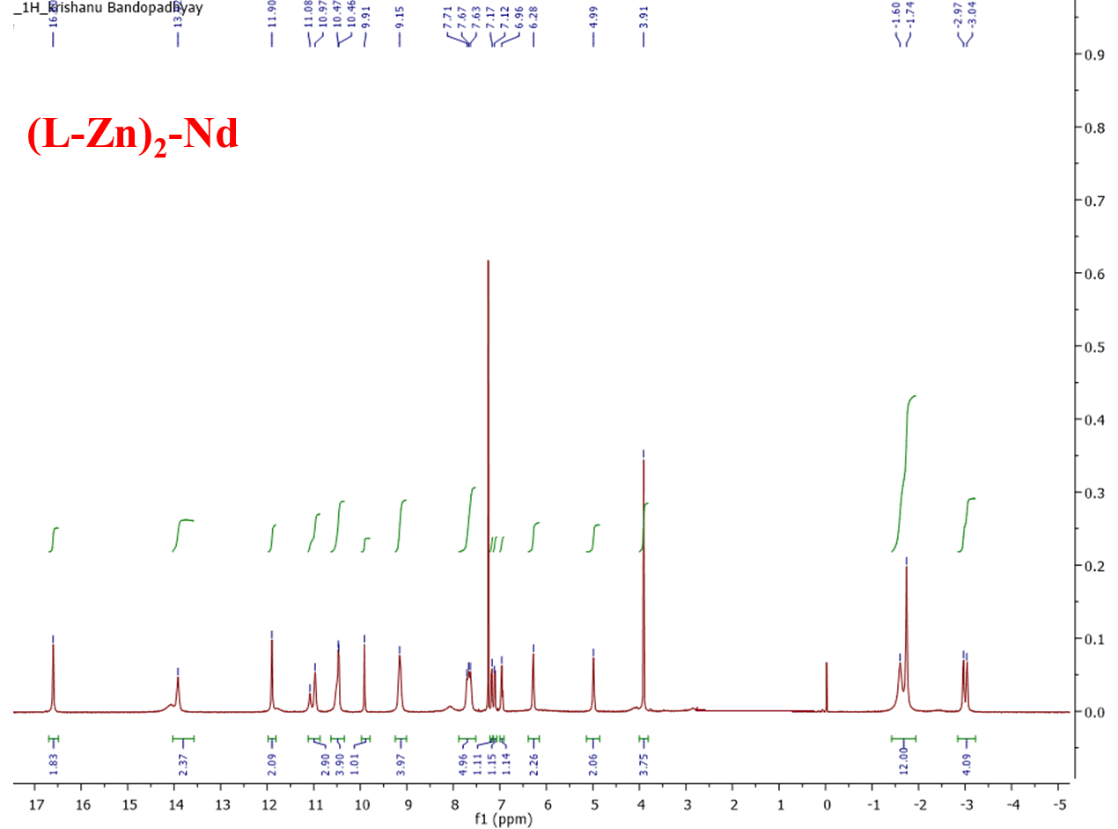 |
| 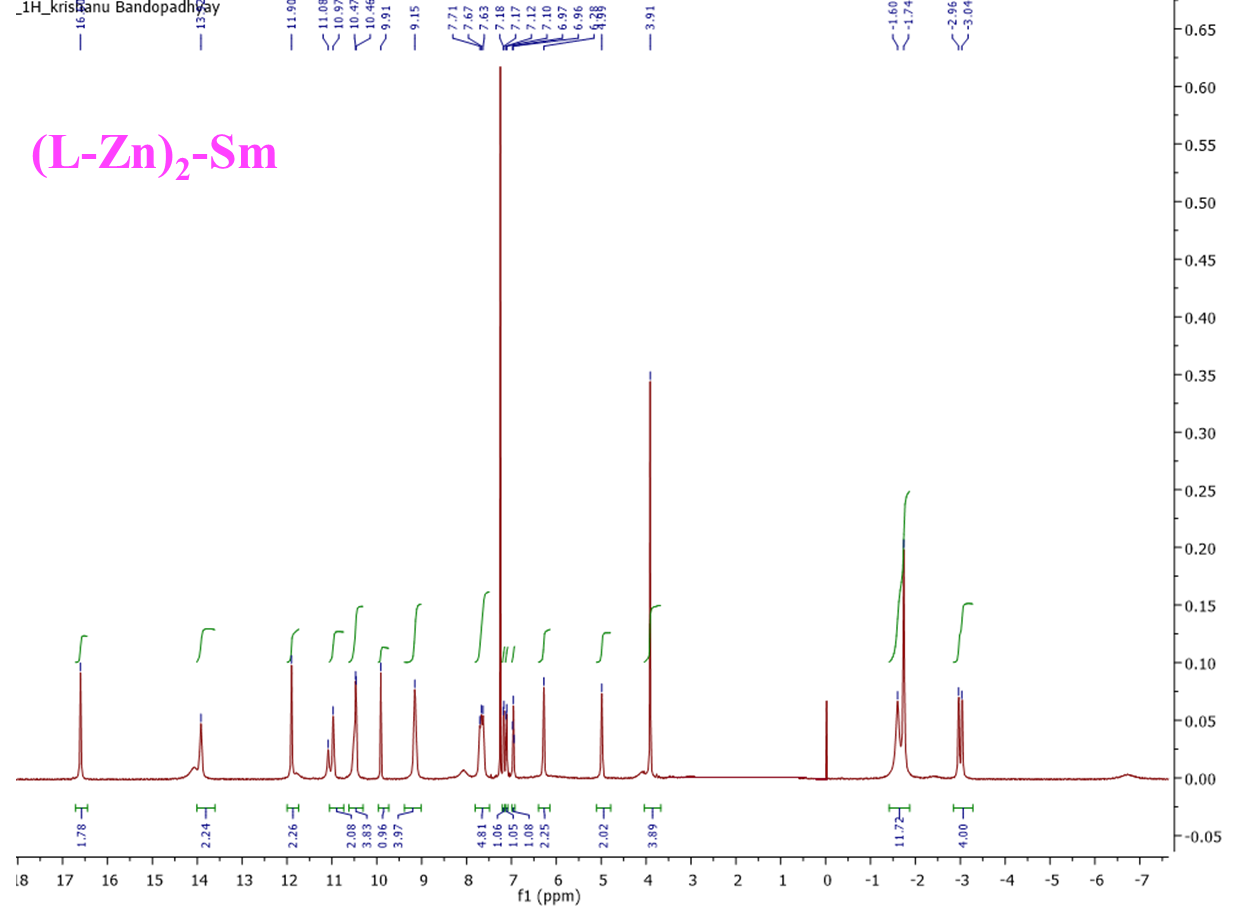 |
| 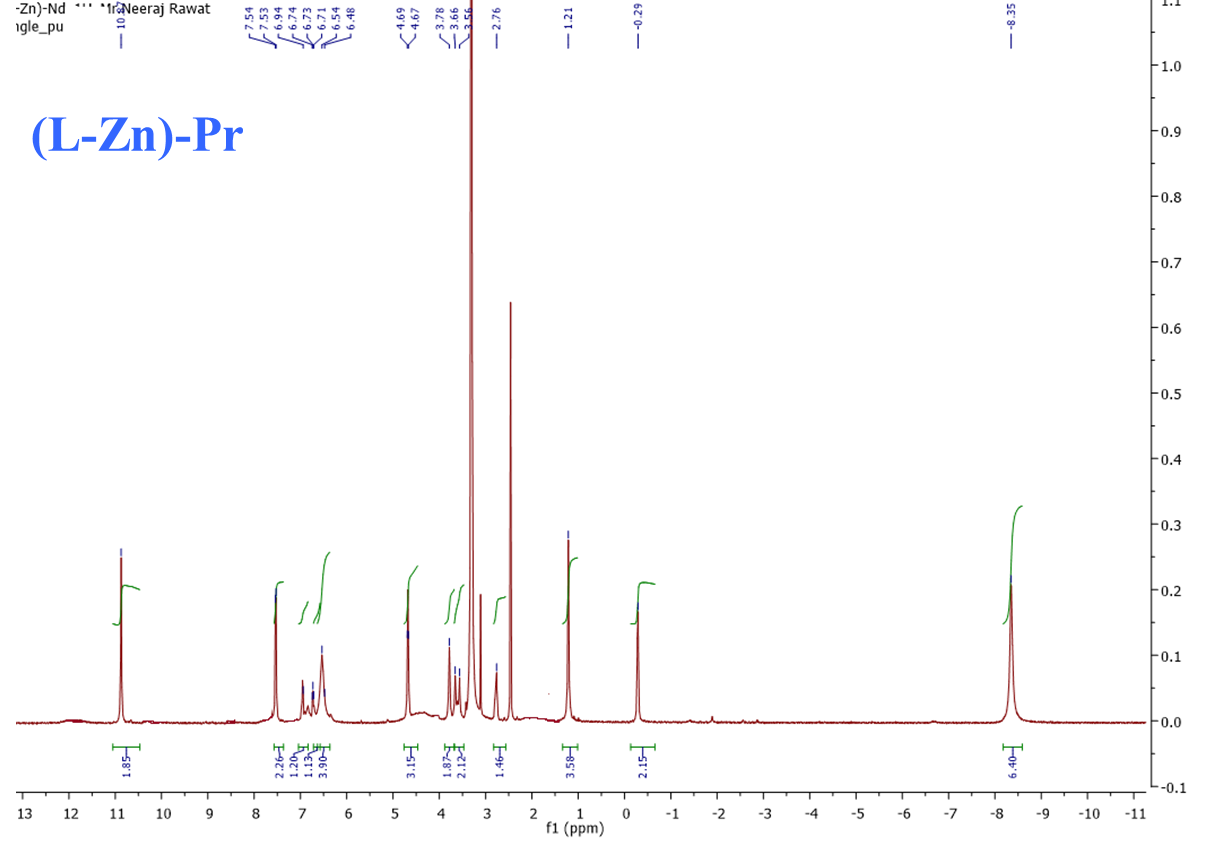 |
| 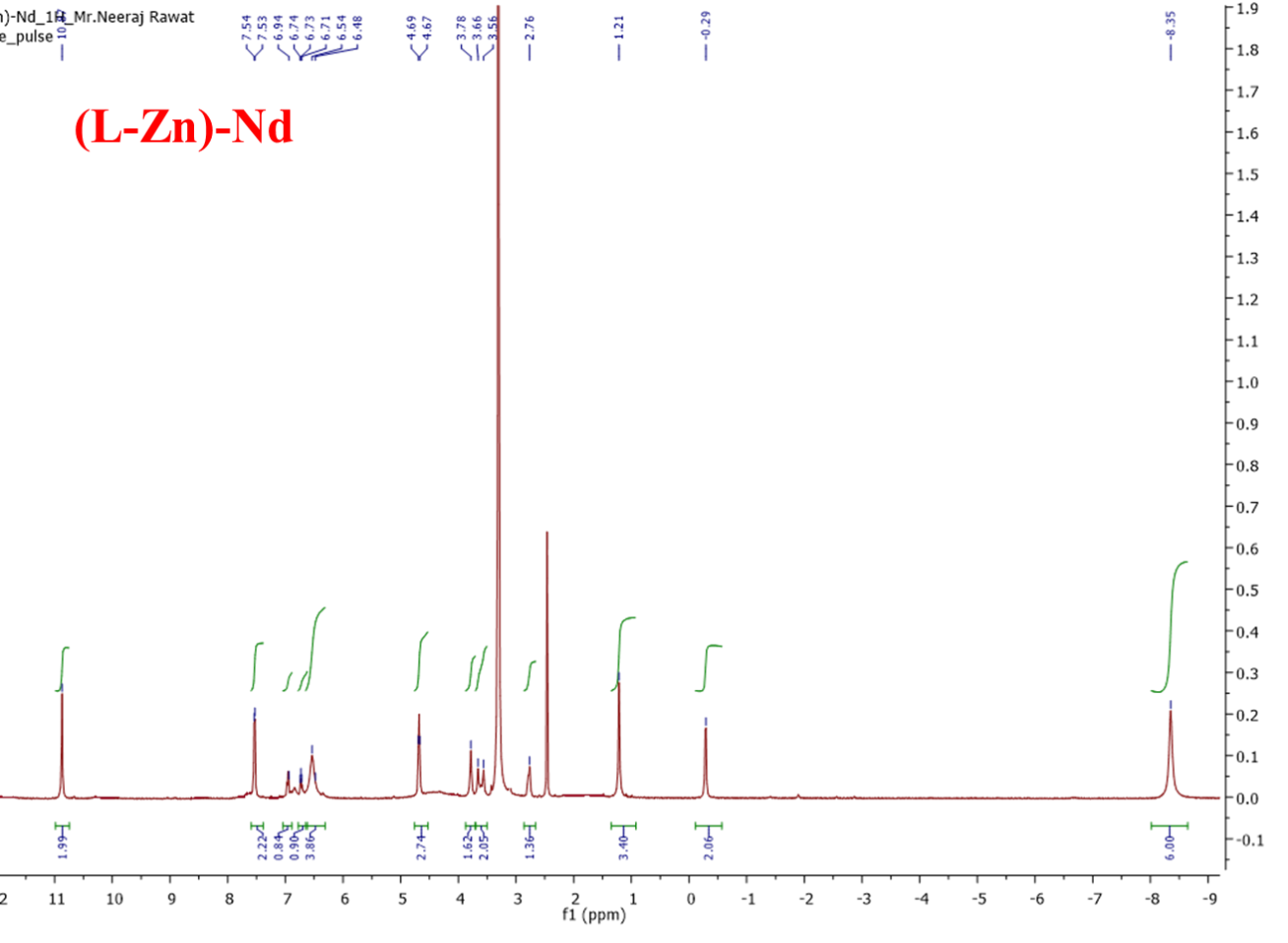 |
| 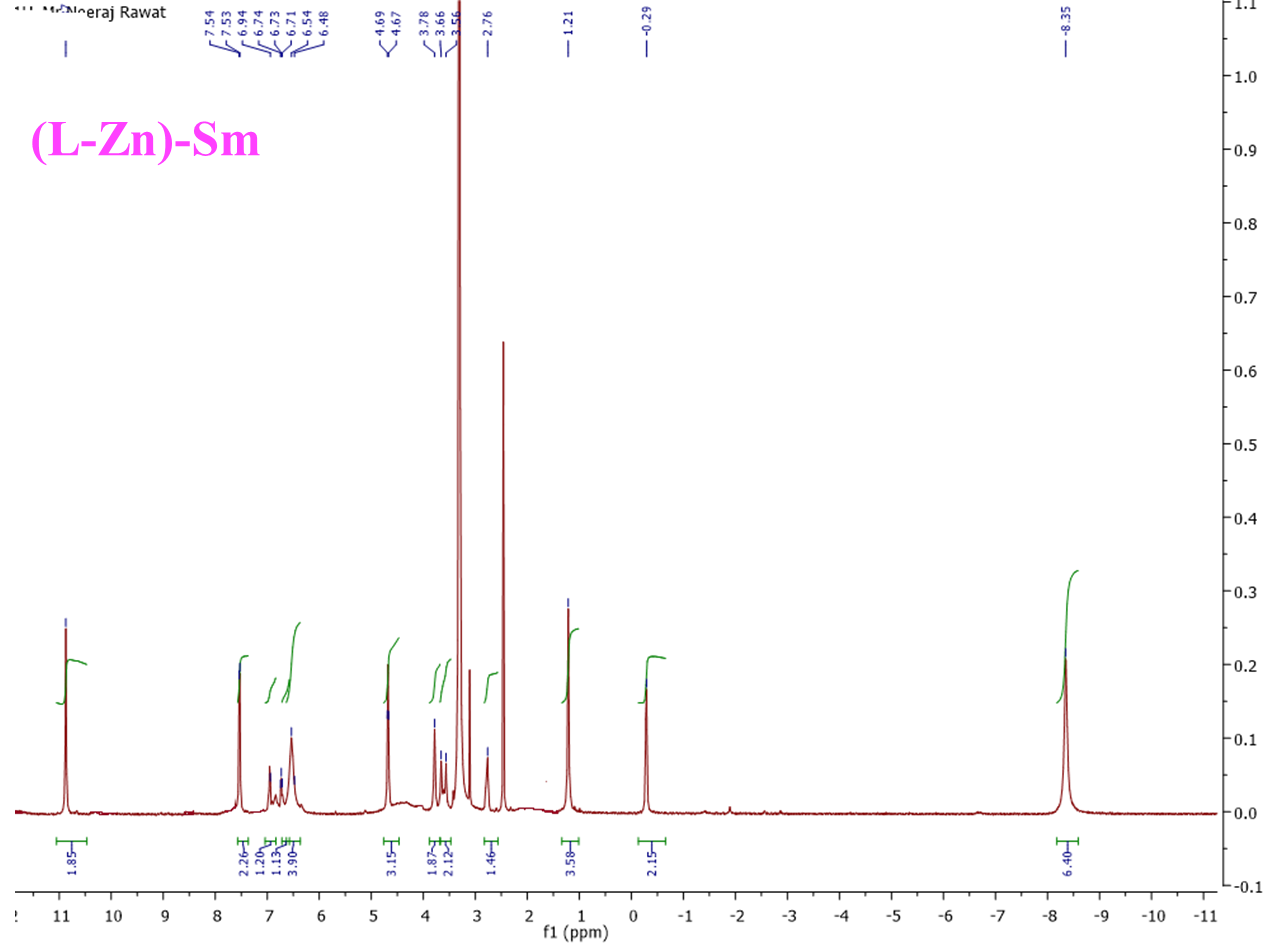 |
| **Figure S1**: ^1^H NMR of the ligands **L**, **[(L-Zn)_2_-Pr]**, **[(L-Zn)_2_-Nd]**, **[(L-Zn)_2_-Sm]** in CDCl_3_, and **[(L-Zn)-Pr]**, **[(L-Zn)-Nd]** and **[(L-Zn)-Sm]** in DMSO-D_6_. |

**^1^HNMR Data:**

**[(L-Zn)_2_-Pr]:** 1H NMR (500 MHz, CHLOROFORM -D ) δ 19.55 (s, 2H), 17.46 (s, 1H), 17.00 (t, *J* = 10.9 Hz, 2H), 13.32 (d, *J* = 135.8 Hz, 2H), 11.97 (s, 2H), 11.72 (d, *J* = 104.7 Hz, 2H), 11.43 (d, *J* = 7.0 Hz, 4H), 11.08 (s, 1H), 10.29 (t, *J* = 11.5 Hz, 2H), 9.91 (s, 1H), 9.31 (s, 5H), 7.18 (d, *J* = 7.3 Hz, 1H), 7.11 (d, *J* = 7.3 Hz, 1H), 6.97 (d, *J* = 7.4 Hz, 1H), 5.08 (d, *J* = 6.2 Hz, 2H), 4.36 – 3.99 (m, 2H), 3.91 (s, 3H), -1.44 (s, 6H), -1.69 (s, 6H), -5.36 (d, *J* = 6.5 Hz, 2H), -6.48 (d, *J* = 4.9 Hz, 2H).

**[(L-Zn)_2_-Nd]:** ^1^H NMR (500 MHz, CHLOROFORM -D) δ 16.60 (s, 2H), 13.92 (s, 2H), 11.90 (s, 2H), 10.97 (s, 2H), 10.47 (d, *J* = 5.9 Hz, 4H), 9.91 (s, 1H), 9.15 (s, 4H), 7.81 – 7.49 (m, 5H), 7.17 (d, *J* = 7.5 Hz, 1H), 7.11 (d, *J* = 7.6 Hz, 1H), 6.96 (t, *J* = 7.8 Hz, 1H), 6.28 (s, 2H), 4.99 (s, 2H), 3.91 (s, 4H), -1.67 (d, *J* = 68.7 Hz, 12H), -3.00 (d, *J* = 36.0 Hz, 4H).

**[(L-Zn)_2_-Sm]:** ^1^H NMR (500 MHz, CHLOROFORM -D) δ 16.60 (s, 2H), 13.92 (s, 2H), 11.90 (s, 2H), 10.97 (s, 2H), 10.47 (d, *J* = 5.9 Hz, 4H), 9.91 (s, 1H), 9.15 (s, 4H), 7.81 – 7.49 (m, 5H), 7.17 (d, *J* = 7.5 Hz, 1H), 7.11 (d, *J* = 7.6 Hz, 1H), 6.96 (t, *J* = 7.8 Hz, 1H), 6.28 (s, 2H), 4.99 (s, 2H), 3.91 (s, 4H), -1.67 (d, *J* = 68.7 Hz, 12H), -3.00 (d, *J* = 36.0 Hz, 4H).

**[(L-Zn)-Pr]:** ^1^H NMR (500 MHz, DMSO-D6 ) δ 10.87 (s, 2H), 7.53 (d, *J* = 7.3 Hz, 2H), 6.94 (s, 1H), 6.71 (s, 1H), 6.51 (d, *J* = 29.6 Hz, 3H), 4.68 (d, *J* = 12.1 Hz, 3H), 3.78 (s, 2H), 3.61 (d, *J* = 47.8 Hz, 2H), 2.76 (s, 1H), 1.25 (s, 3H), -0.29 (s, 2H), -8.35 (s, 6H).

**[(L-Zn)-Nd]:** ^1^H NMR (500 MHz, DMSO-D6) δ 10.85 (s, 2H), 7.52 (d, *J* = 7.3 Hz, 2H), 6.90 (s, 1H), 6.71 (s, 1H), 6.49 (d, *J* = 29.6 Hz, 3H), 4.64 (d, *J* = 12.1 Hz, 3H), 3.78 (s, 2H), 3.65 (d, *J* = 47.8 Hz, 2H), 2.76 (s, 1H), 1.21 (s, 3H), -0.27 (s, 2H), -8.38 (s, 6H).

**[(L-Zn)-Sm]:** ^1^H NMR (500 MHz, DMSO-D6) δ 10.92 (s, 2H), 7.56 (d, *J* = 7.3 Hz, 2H), 6.89 (s, 1H), 6.73 (s, 1H), 6.54 (d, *J* = 29.6 Hz, 3H), 4.68 (d, *J* = 12.1 Hz, 3H), 3.87 (s, 2H), 3.61 (d, *J* = 47.8 Hz, 2H), 2.77 (s, 1H), 1.26 (s, 3H), -0.25 (s, 2H), -8.20 (s, 6H).

**FTIR studies**:

| 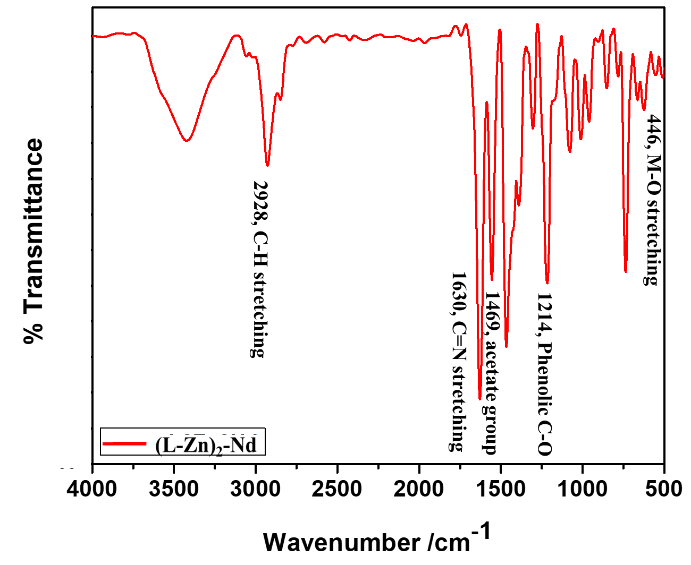 | 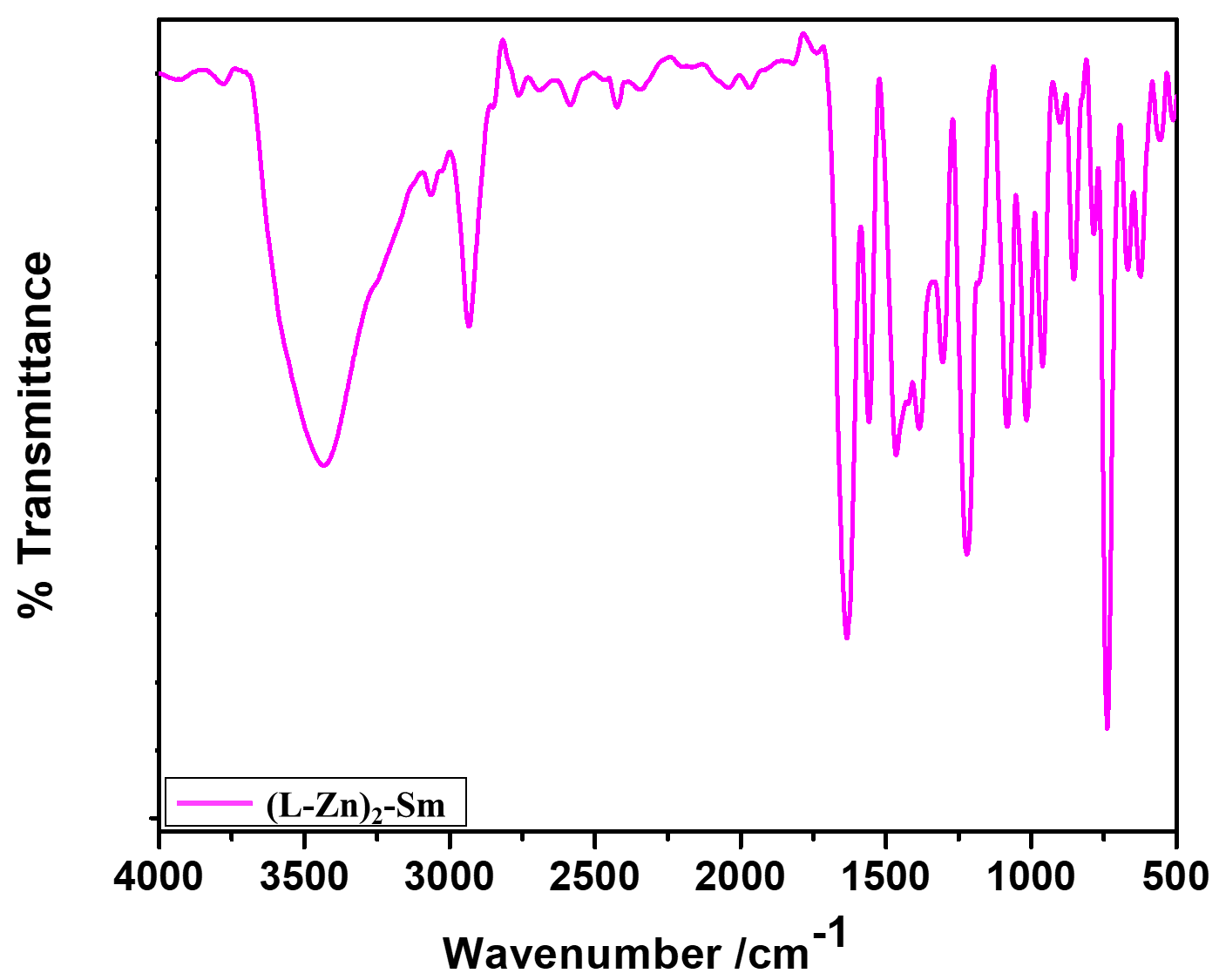 |
| --- | --- |
| 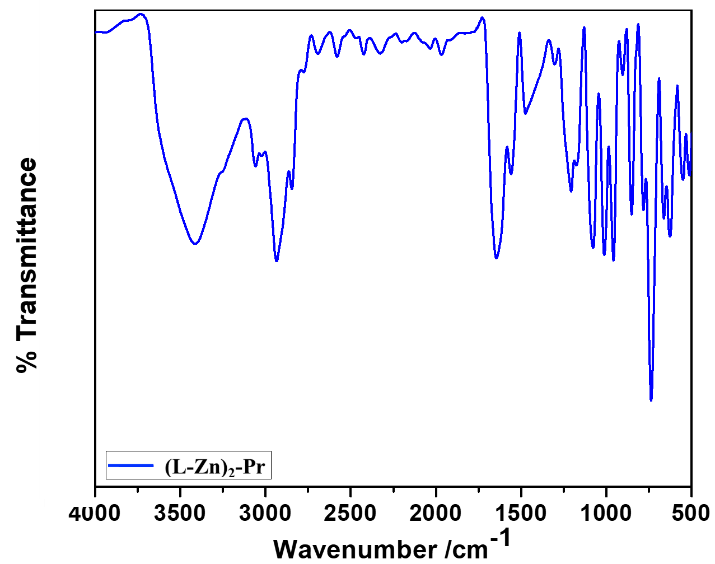 | 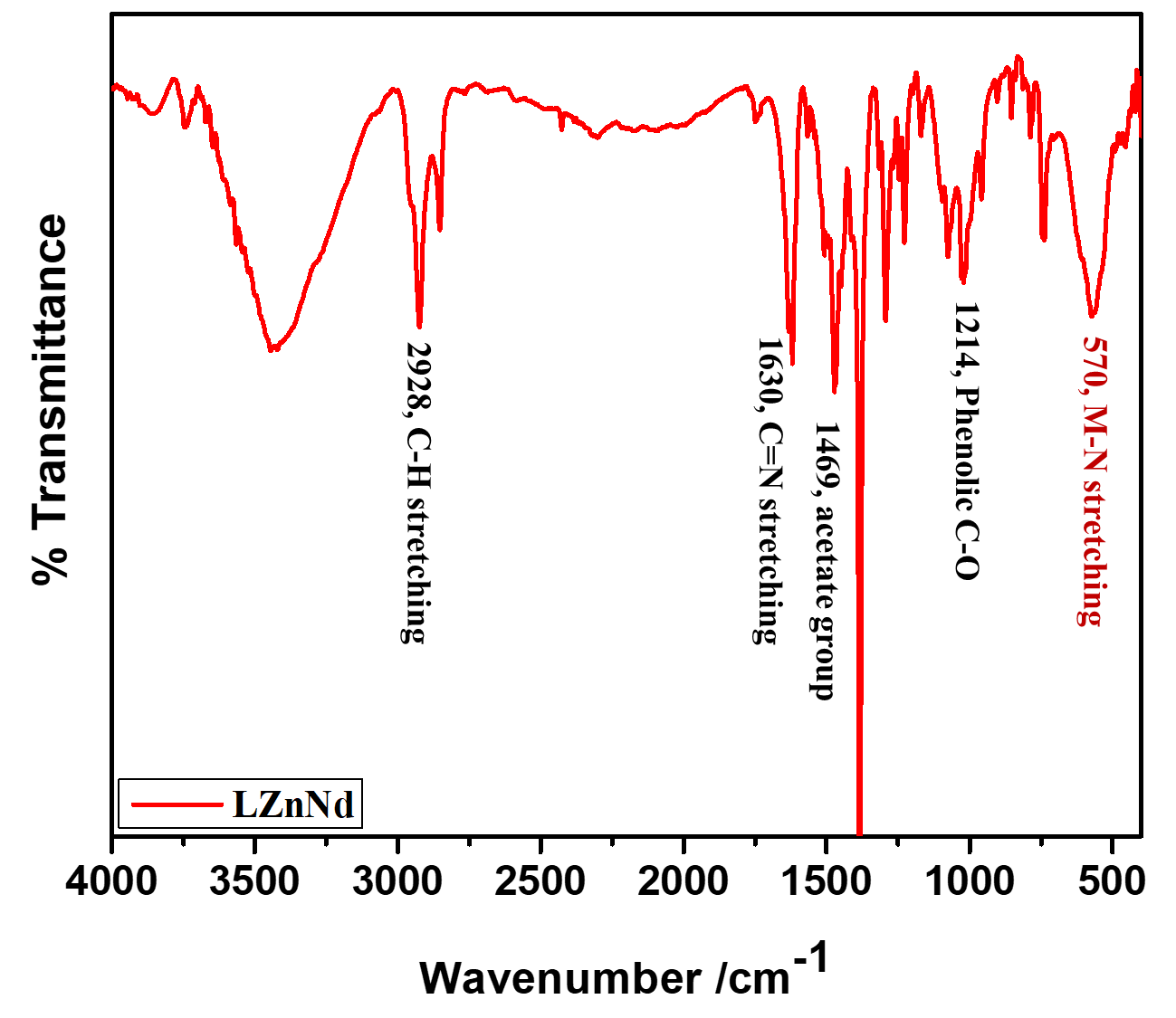 |
| 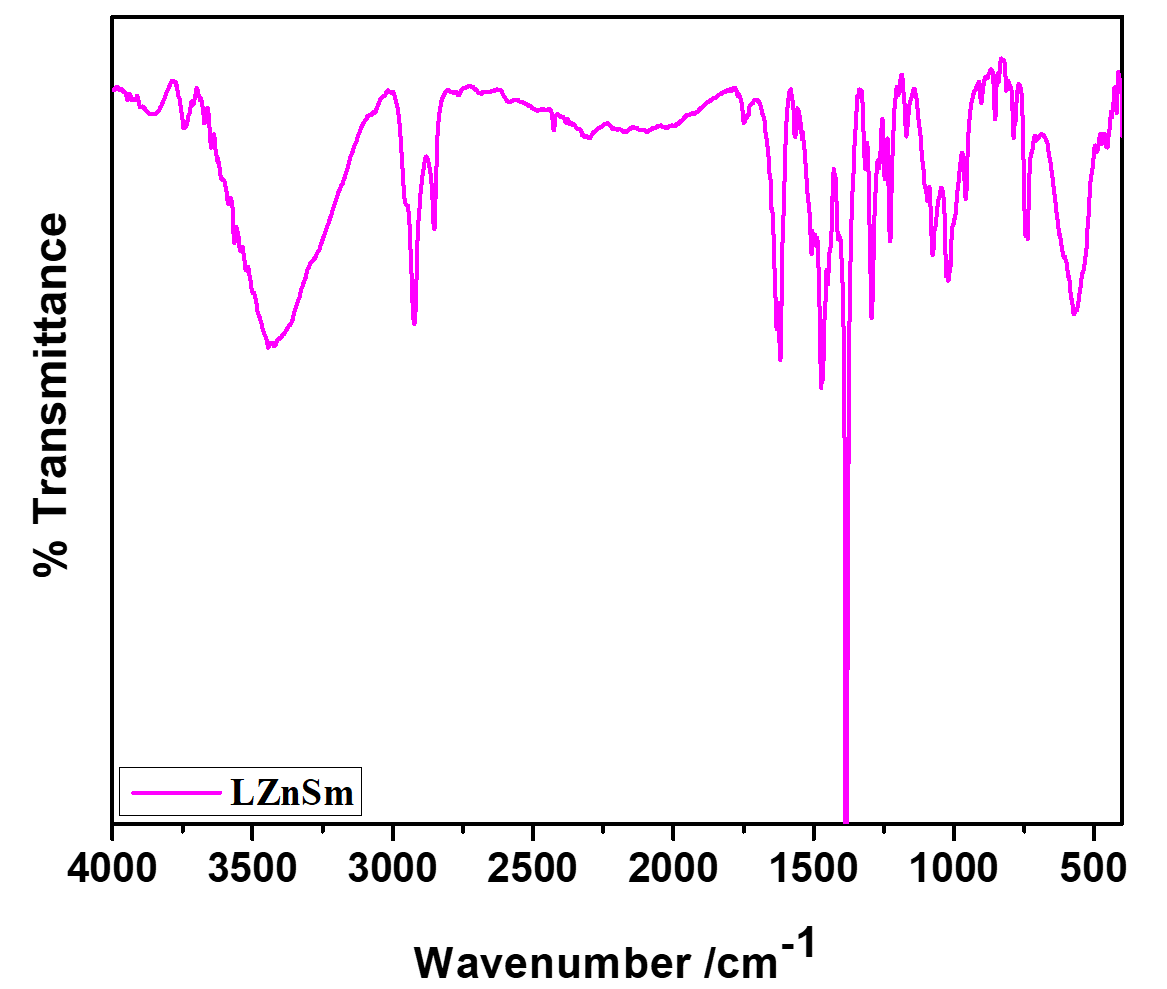 | 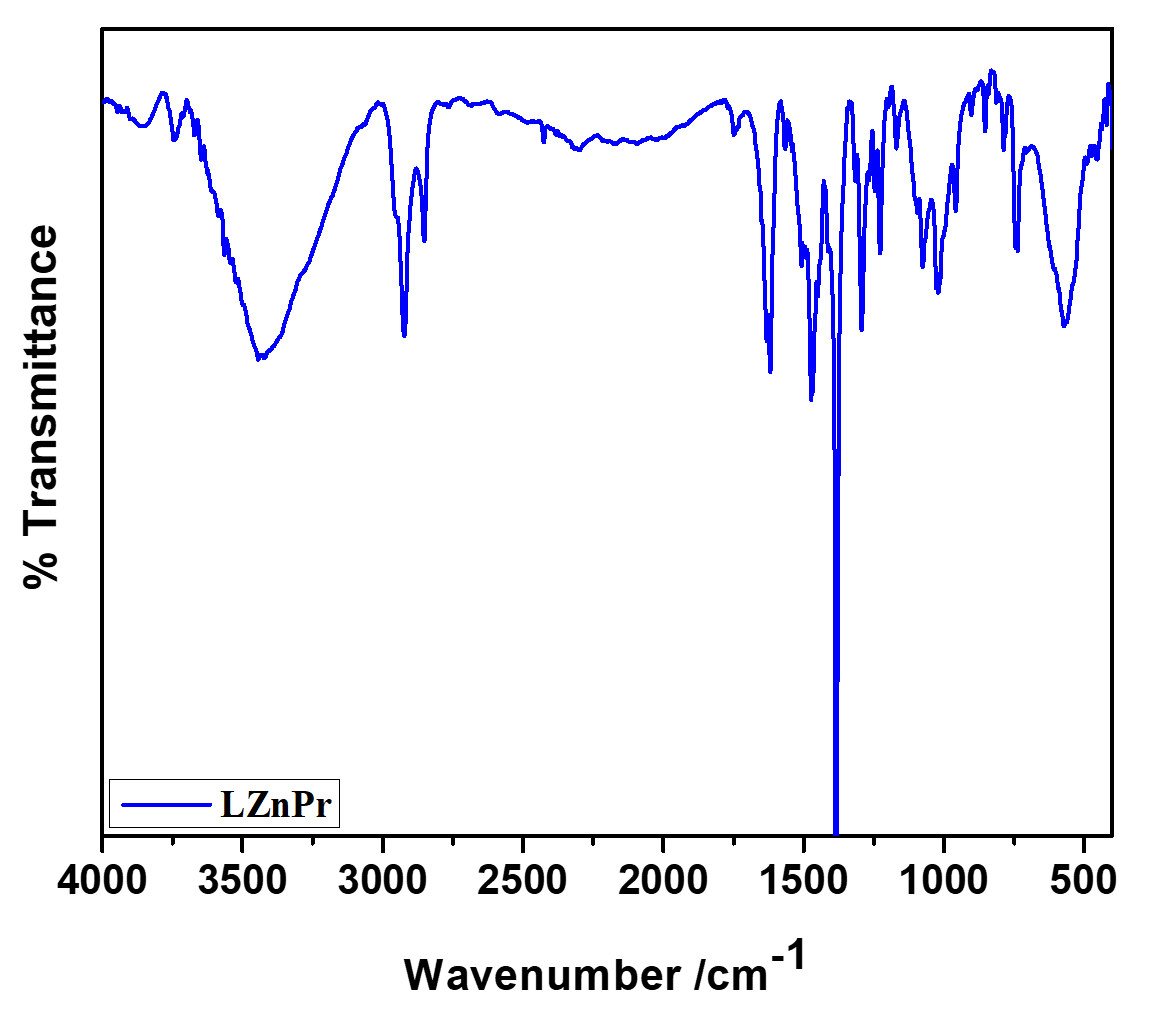 |
| 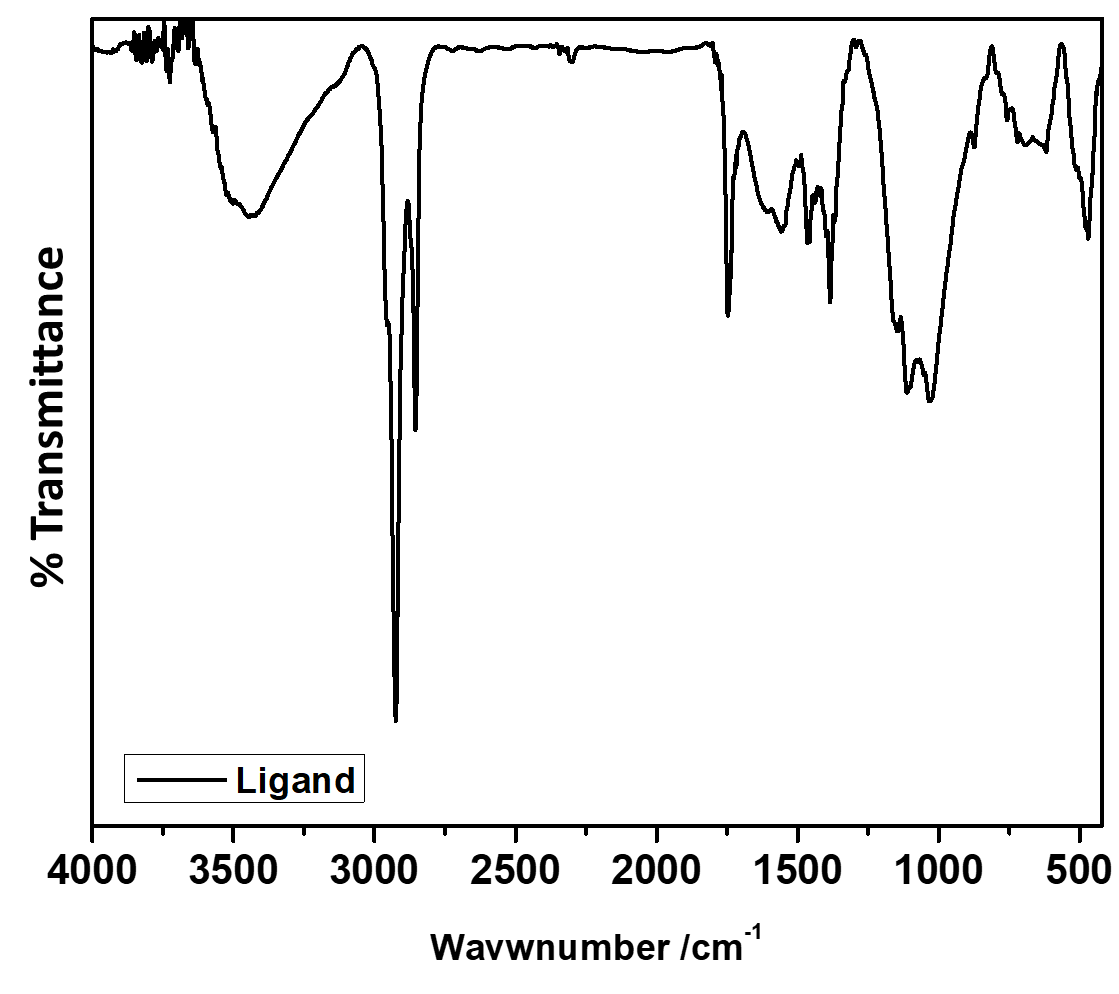 | |
| **Figure S2**: FTIR spectra of heterotrimetallic **[L_2_-Zn_2_-Ln]** and heterobimetallic **[L-Zn-Ln]** complexes. | |

**PXRD studies:**

| 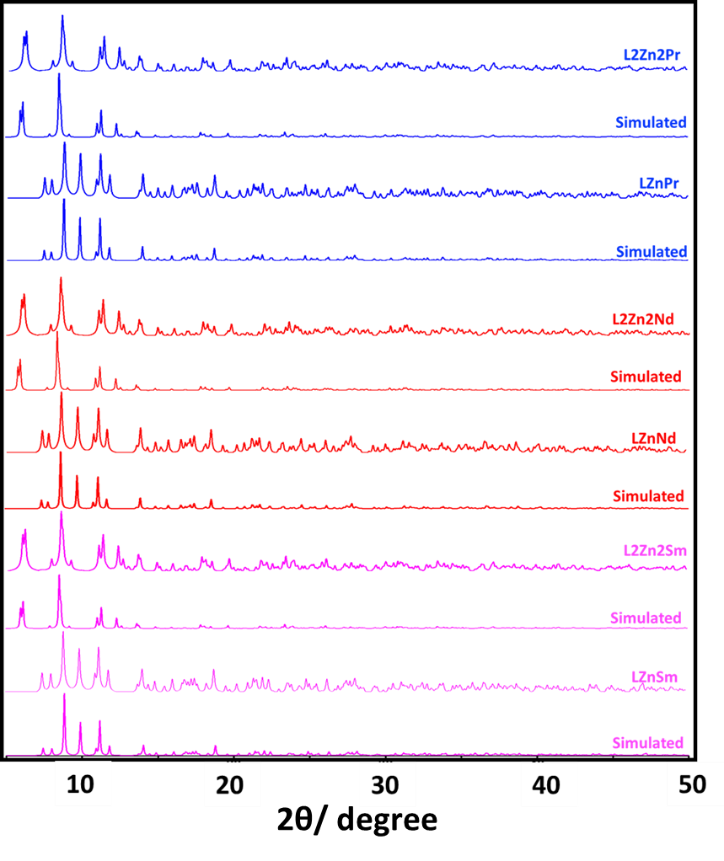 |
| --- |
| **Figure S3**: PXRD patterns for the complexes (a) [(L–Zn)–Ln] and [(L–Zn)_2_–Ln]. For comparison, the simulated patterns generated from SCXRD data are also given. |
|  |

**
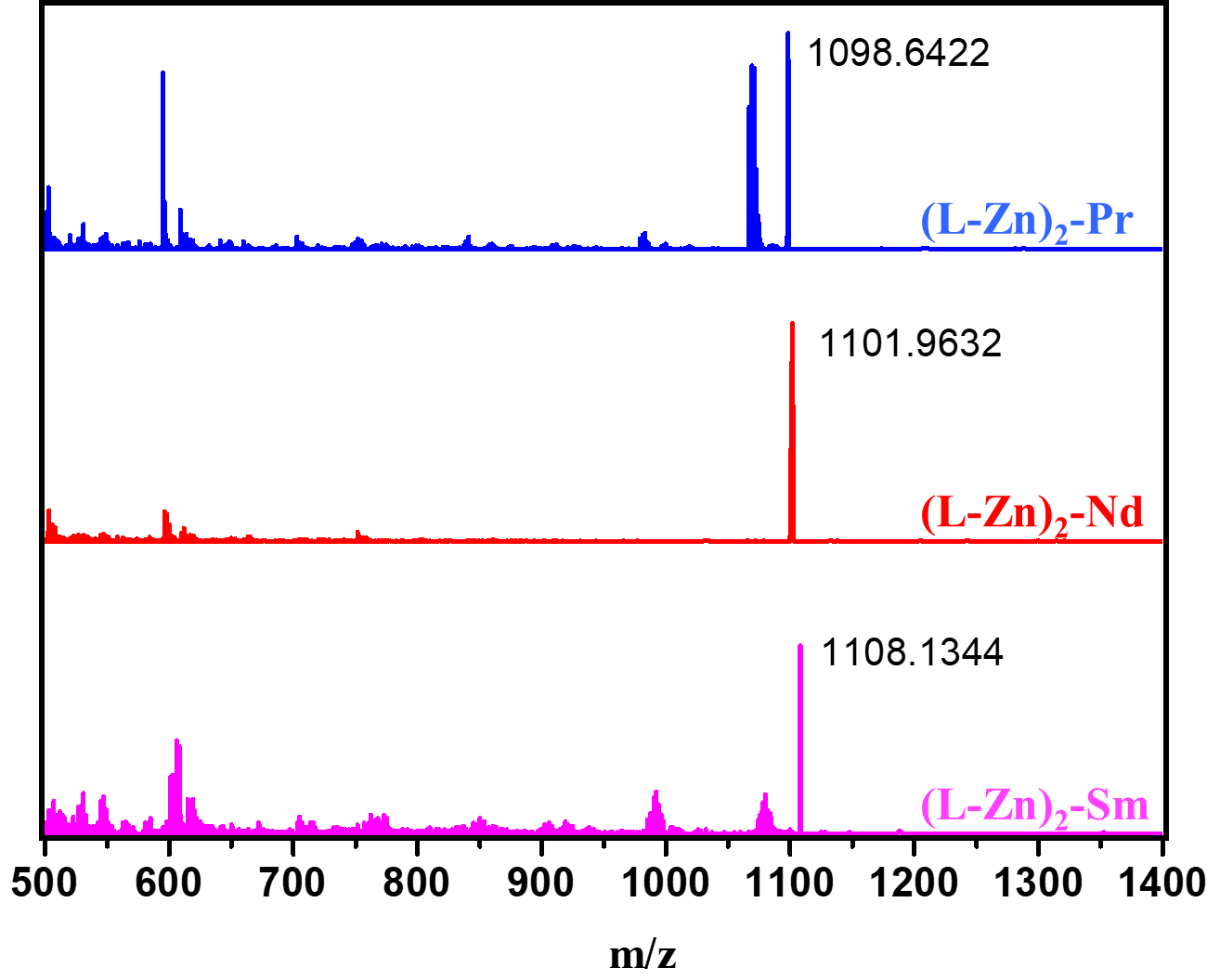

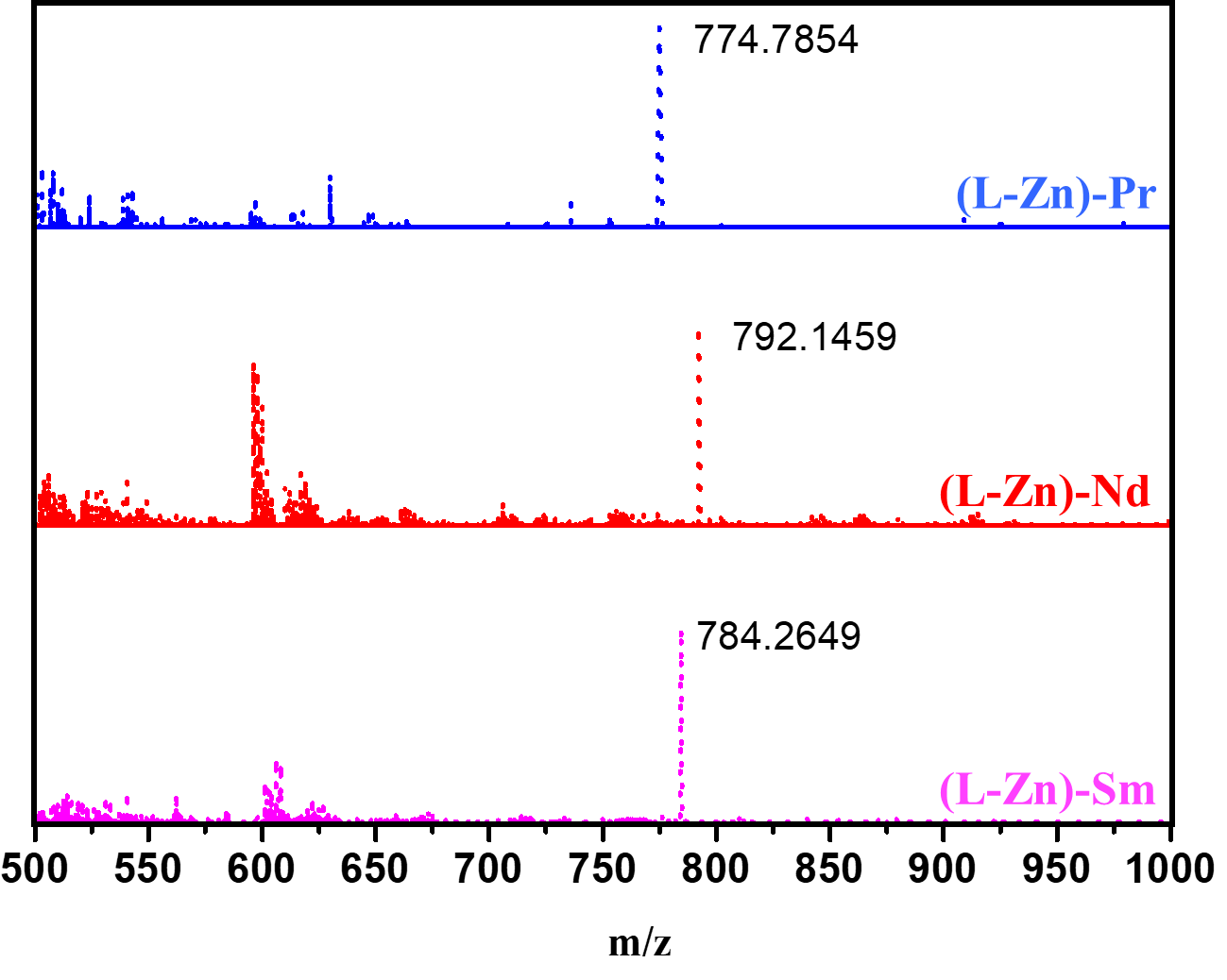
HRMS studies:**

|  |  |
| --- | --- |
| **Figure S4:** HRMS spectrum of bimetallic and trimetallic complexes. HRMS (positive mode) of trimetallic complexes showing the **[M–NO_3_]^+^** ion peak due to the loss of the nitrate anion upon ionization, whereas the bimetallic complex shows **[M]^+^** as a molecular ion peak, as it does not content any anion counterpart. | |

**Check-CIF report:**

**(L-Zn)_2_-Pr:**

| 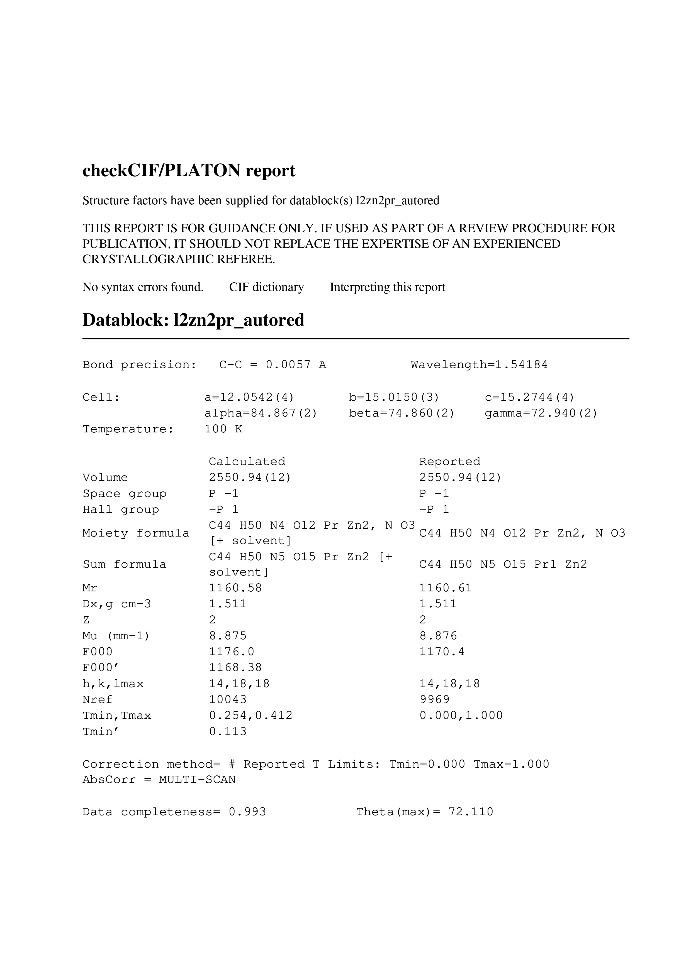 | 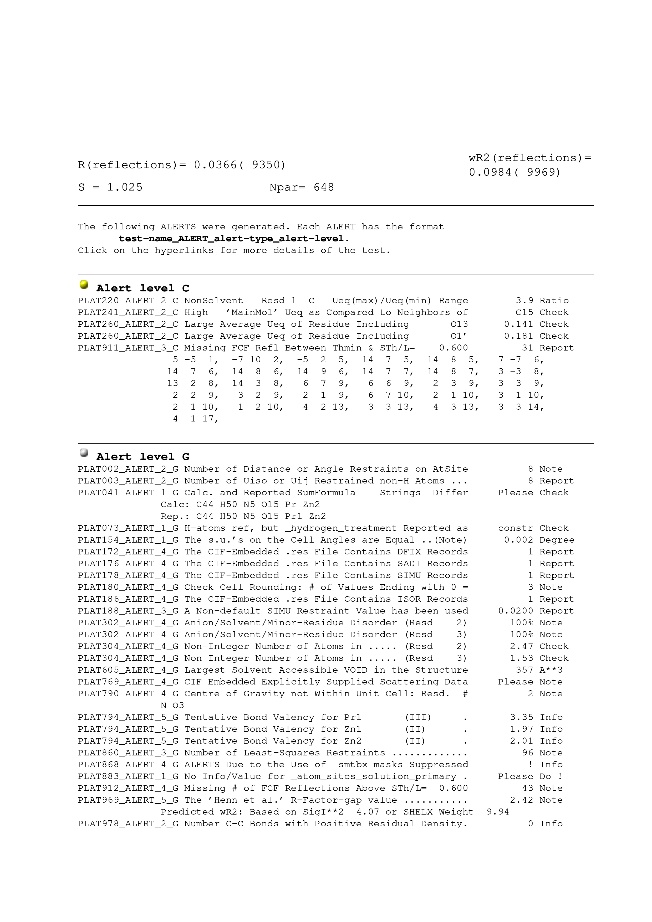 |
| --- | --- |
| 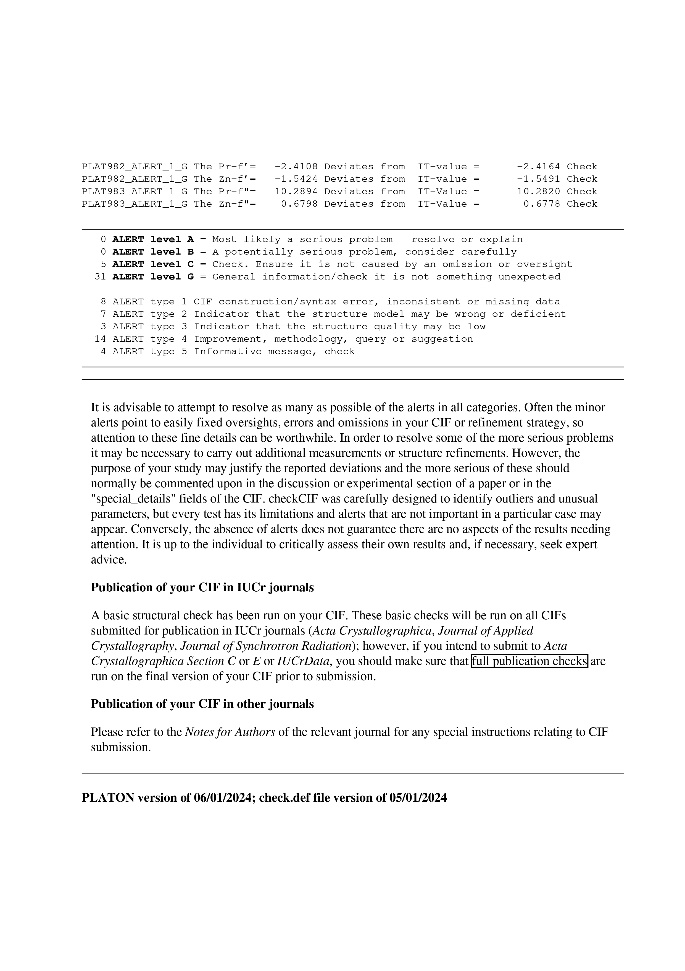 | 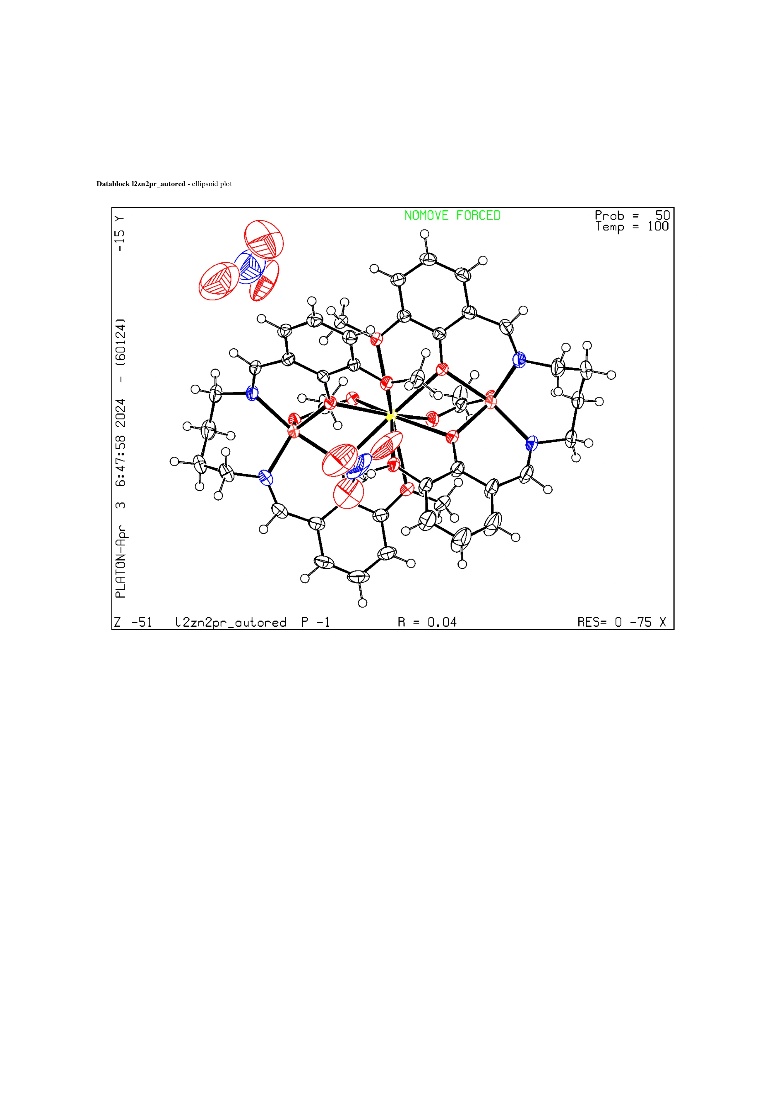 |

**(L-Zn)_2_-Nd:**

| 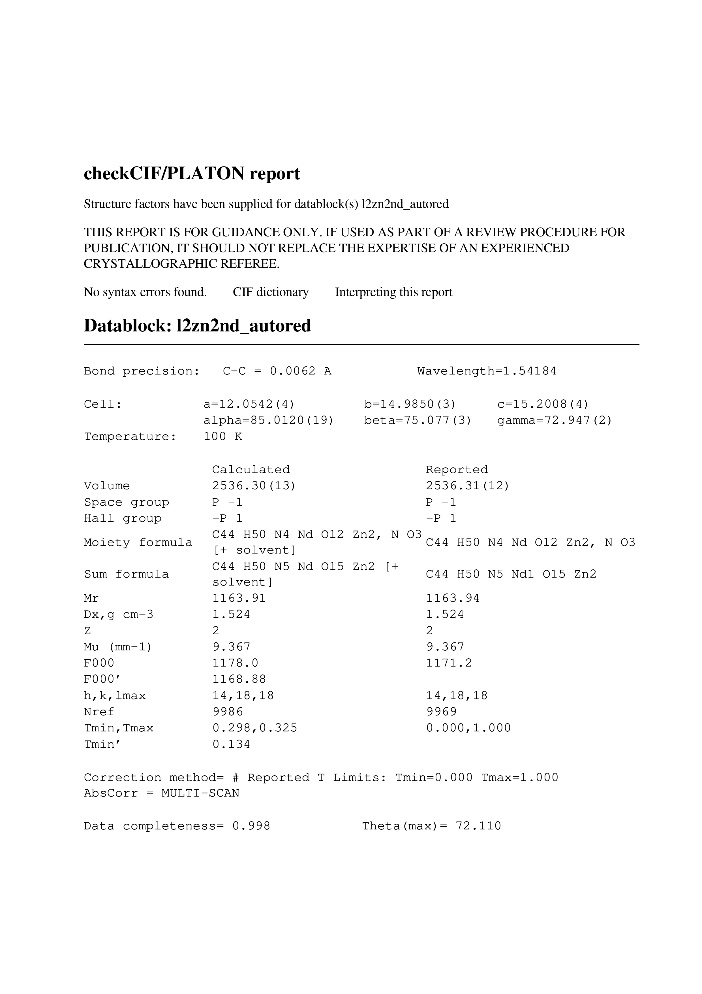 | 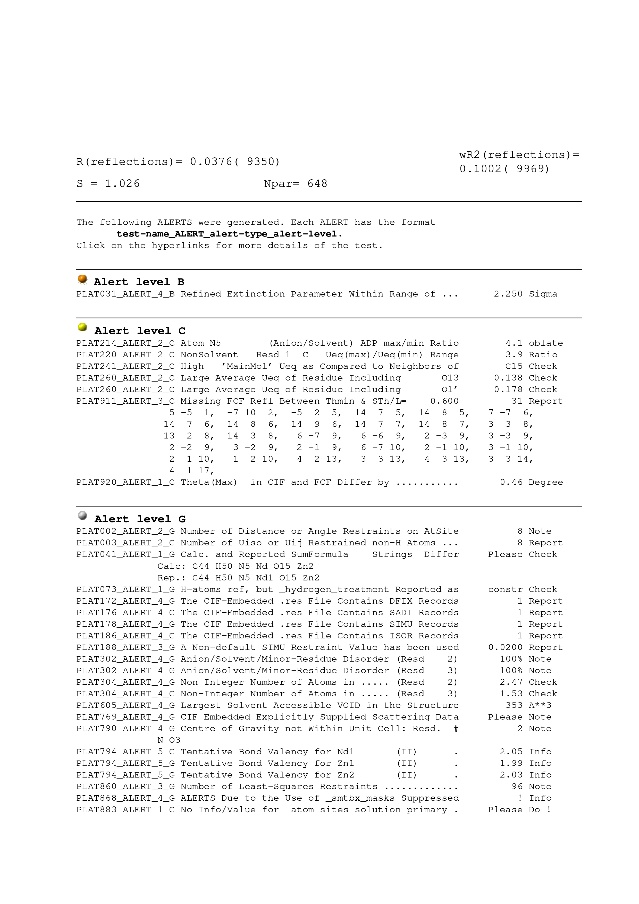 |
| --- | --- |
| 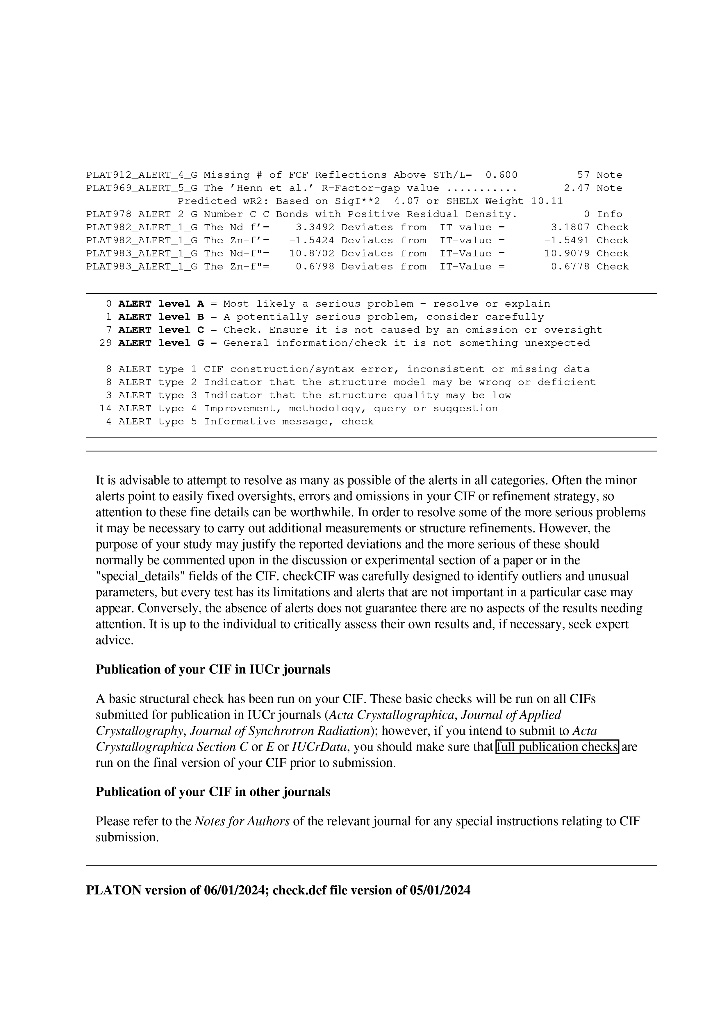 | 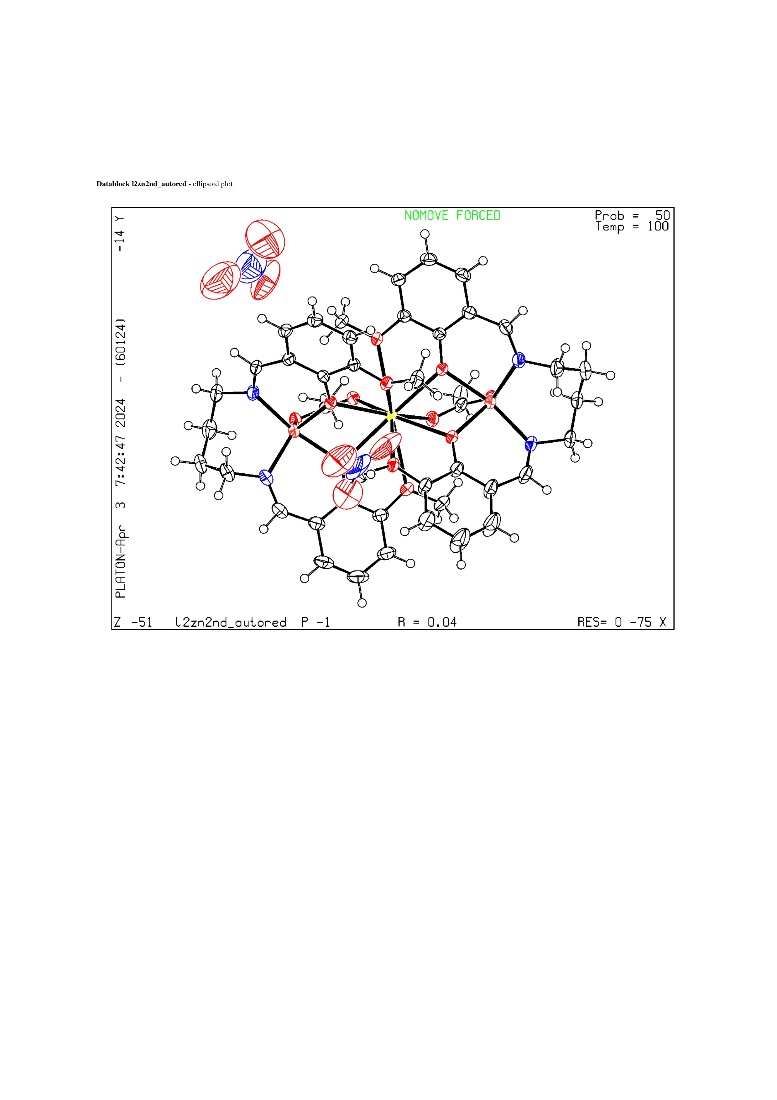 |

**L-Zn-Sm:**

| 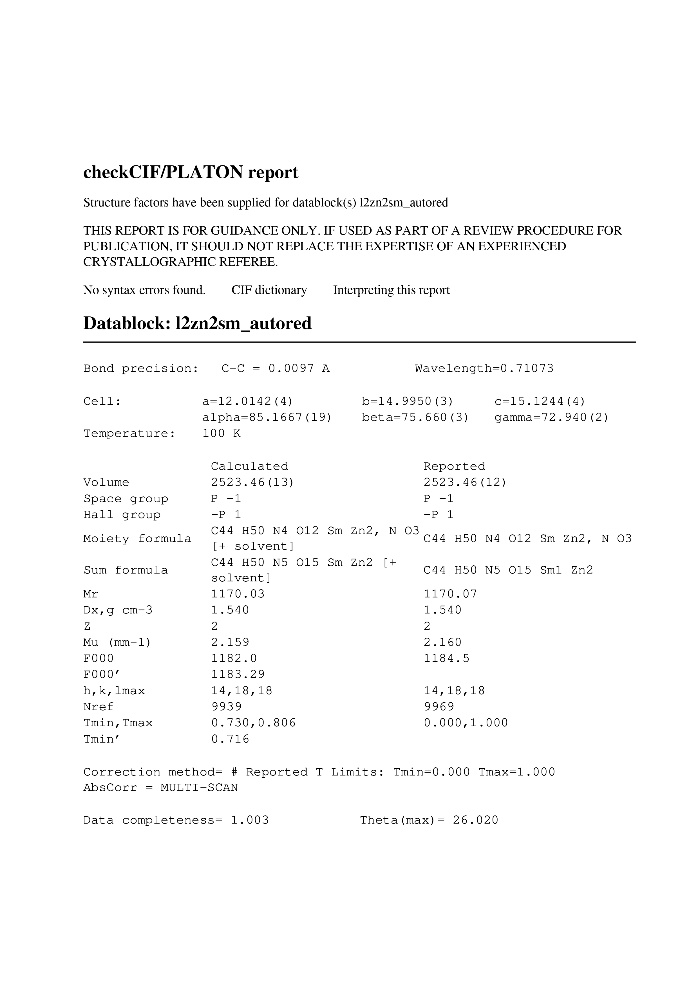 | 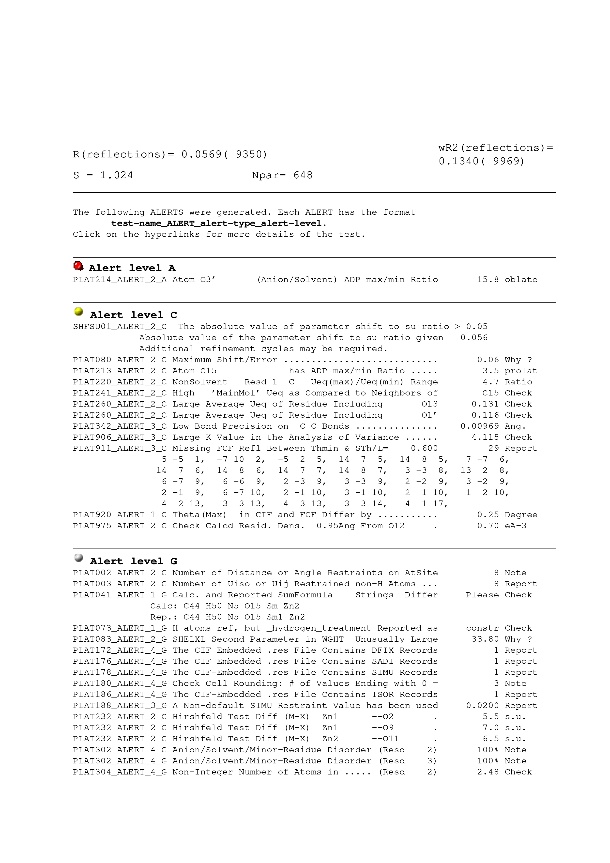 |
| --- | --- |
| 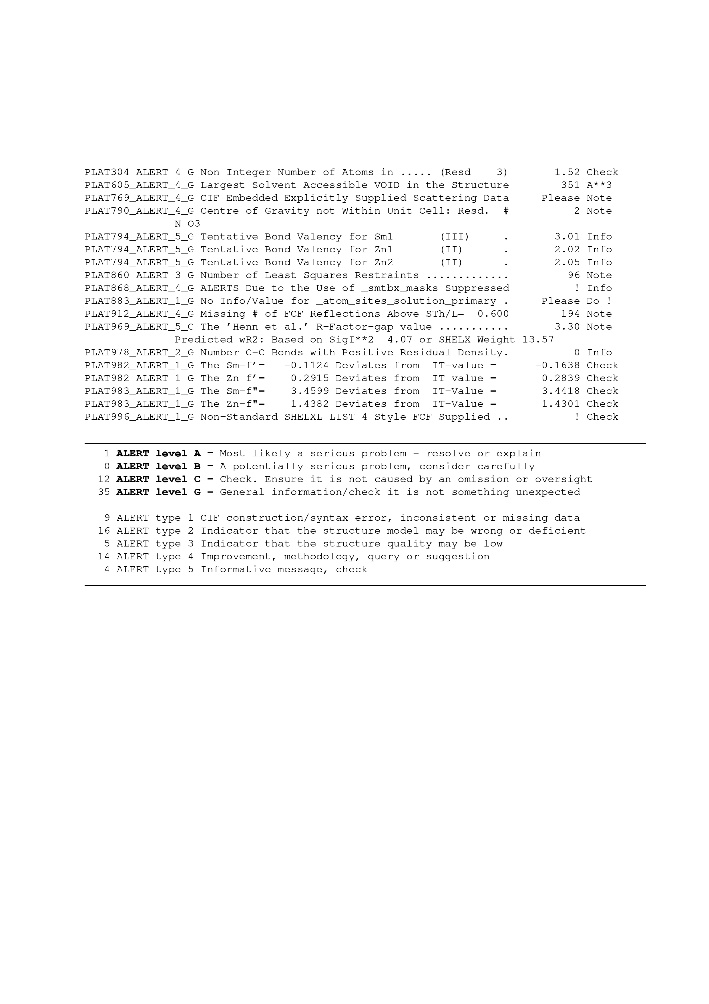 | 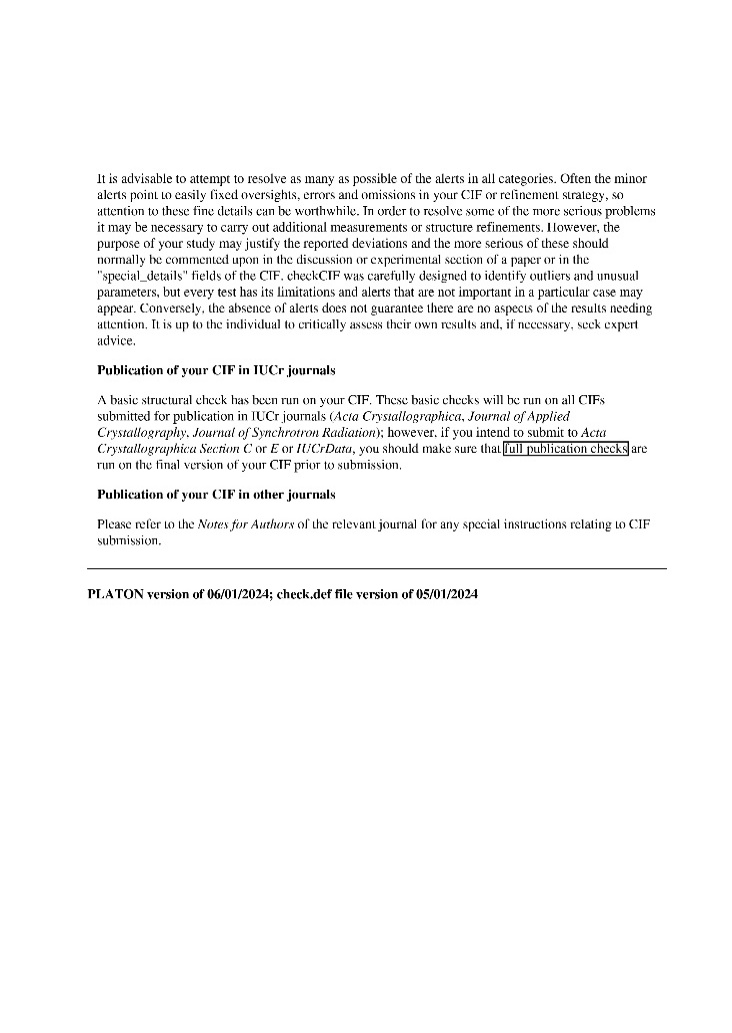 |
| 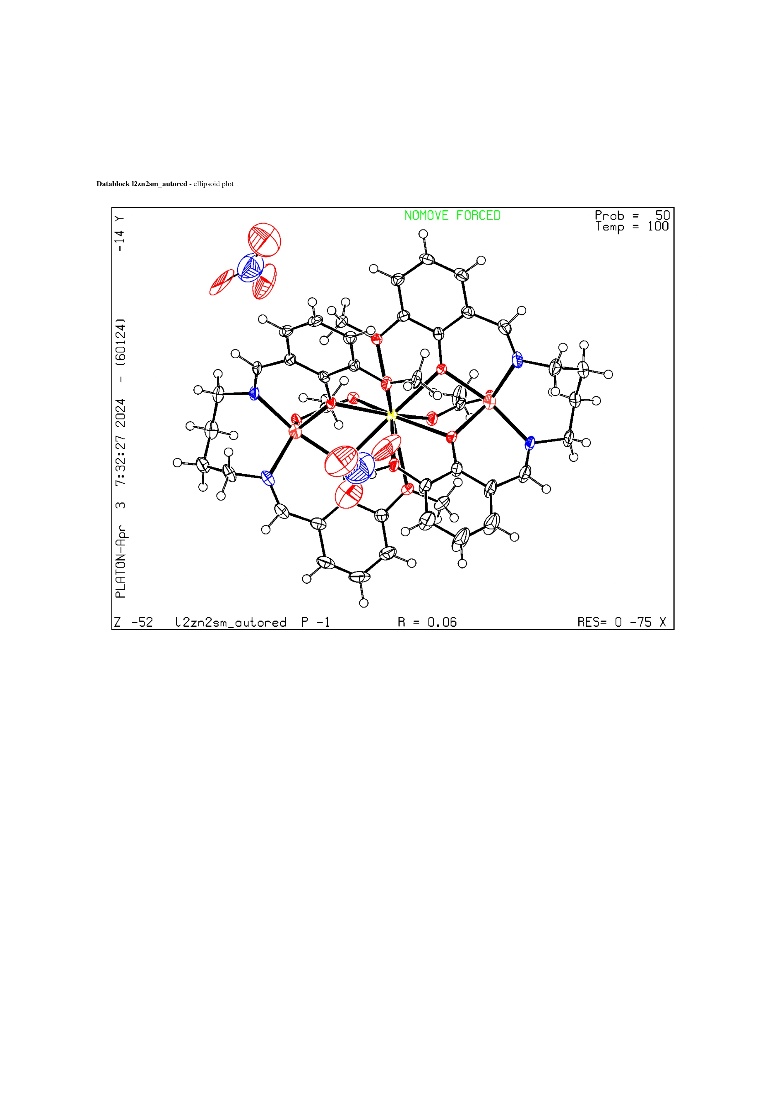 | |

**Crystal packing:**

| 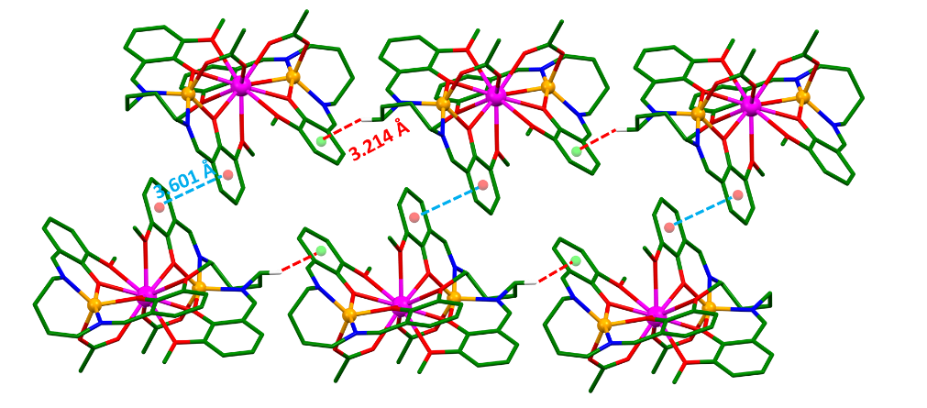  **(a)** |
| --- |
| 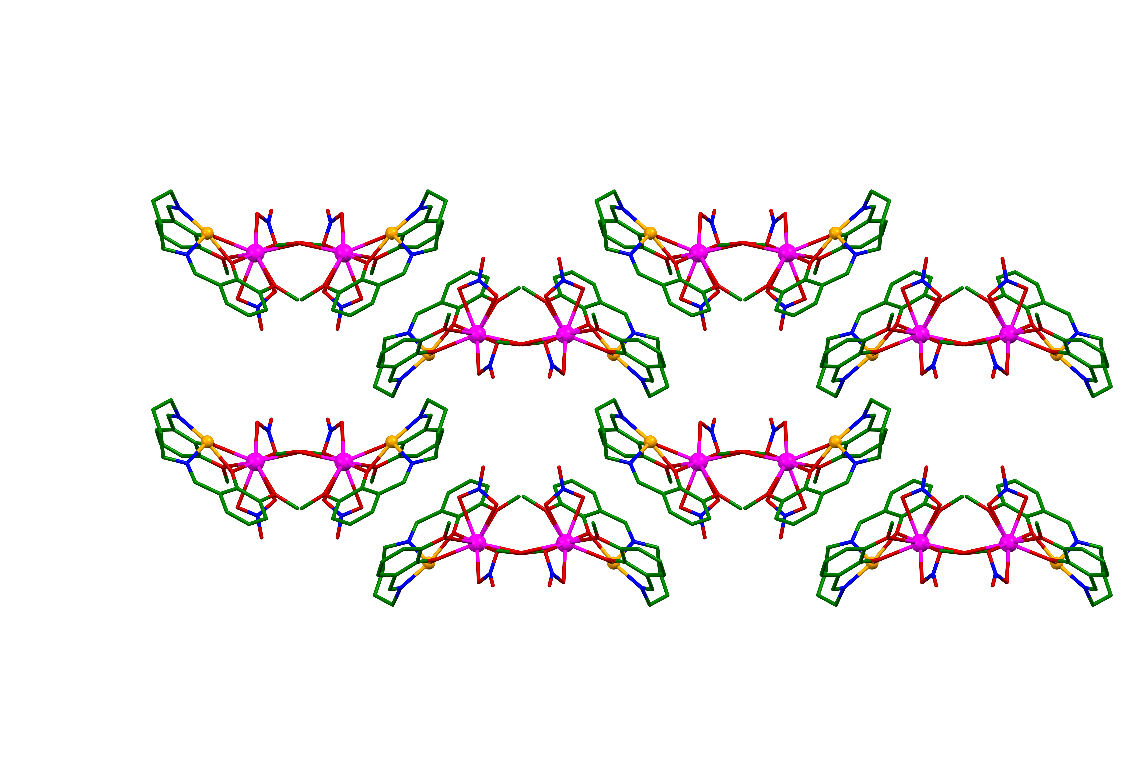  **(b)** |
| **Figure S5:** Important interaction and packing diagram for (a) **[(L−Zn)_2_−Ln]** showing π --- π , C−H--- π and C−H---O interactions and (b) **[(L−Zn)−Ln]** showing C−H--- π and two C−H---O interactions. |

|  |  |
| --- | --- |
| s |  |
| **Figure S6**. X-ray photoelectron spectroscopy C1s, O1s, Zn2p^3/2^, Zn 2p^1/2^, Sm3d^5/2^, and Sm 3d^3/2^ spectrum of the complexes. [L-Zn-Sm] and. [(L-Zn)_2_-Sm] is presented in blue and red, respectively. | |

| **Table S1** The binding energy values and important parameters derived from the X-ray photoelectron studies of [(L-Zn)-Sm] and [(L-Zn)_2_-Sm] complexes | | | | | | | | |
| --- | --- | --- | --- | --- | --- | --- | --- | --- |
| **S.**  **No.** | **Element** | **Transition** | **Peak Energy (eV)** | **FWHM** | | **Peak area** | | **Peak assignment** |
|  |  |  |  | **[(L-Zn)-Sm]** | **[(L-Zn)_2_-Sm]** | **[(L-Zn)-Sm]** | **[(L-Zn)_2_-Sm]** |  |
| 1. | **C** | **1s** | 283.3 | 2.2327 | 1.3323 | 9212 | 9789 | C(sp^2^) |
| 2. |  |  | 286.4 | 4.0812 | 1.9682 | 2367 | 3456 | C-O |
| 3. | **N** | **1s** | 397.2 | 2.1959 | 2.3465 | 6833 | 3458 | N=C |
| 4. |  |  | 400.1 | 0.9321 | - | 5063 | - | N-M |
| 5. |  |  | 404.7 | 0.8321 | 0.5245 | 985 | 486 | N-O |
| 6. | **O** | **1s** | 529.7 | 2.4832 | 2.3968 | 6241 | 6983 | M-O |
| 7. | **Zn** | **2p^3/2^** | 1019.6 | 1.7750 | 2.3901 | 1521 | 1781 | Zn-O |
| 8. |  | **2p^1/2^** | 1042.4 | 1.9973 | 2.5002 | 834 | 858 | Zn-N |
| 9. | **Sm** | **3d^5/2^** | 1081 | 3.7626 | 3.7630 | 1887 | 1594 | Sm-O |
| 10 |  | **3d^3/2^** | 1107.5 | 6.8050 | 8.6058 | 2132 | 1825 | Sm-O |

| 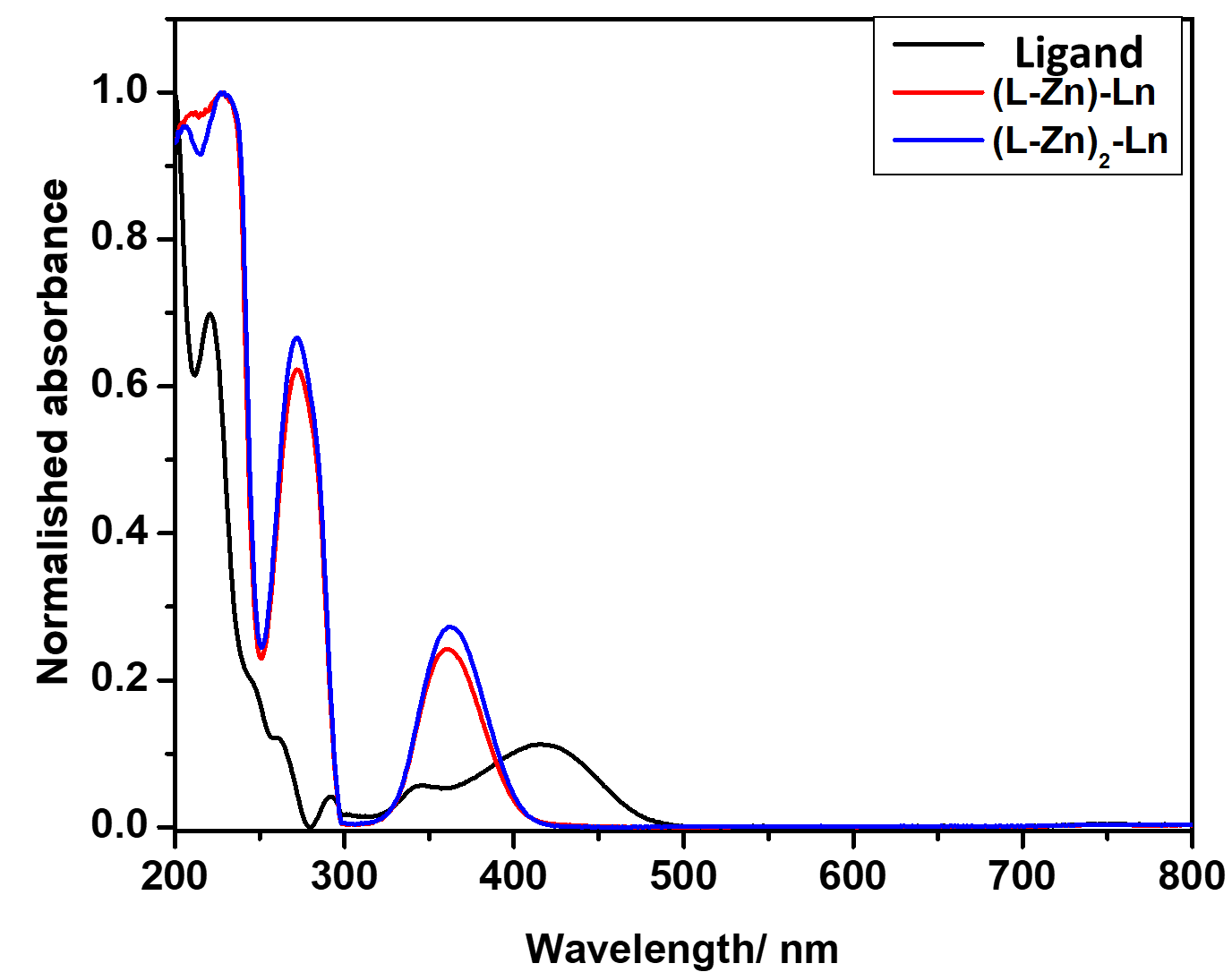 | 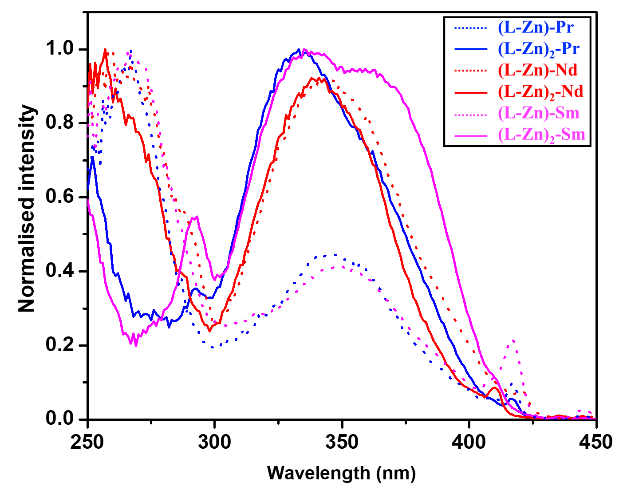  **(b)** |
| --- | --- |
| **Figure S7:** Full range (a) UV-visible absorbance and (b) Excitation spectra of ligand, bimetallic and trimetallic complexes. | |

**(a)**

| **Table S2**. Increase in Luminescence intensity (times) for the Lanthanide Complexes **[(L-Zn)_2_-Ln]** and **[(L-Zn)-Ln]** in methanol. (Petri dish for change in luminescence intensity in the solid state, and test tube for change in luminescence intensity in solution. |
| --- |
| 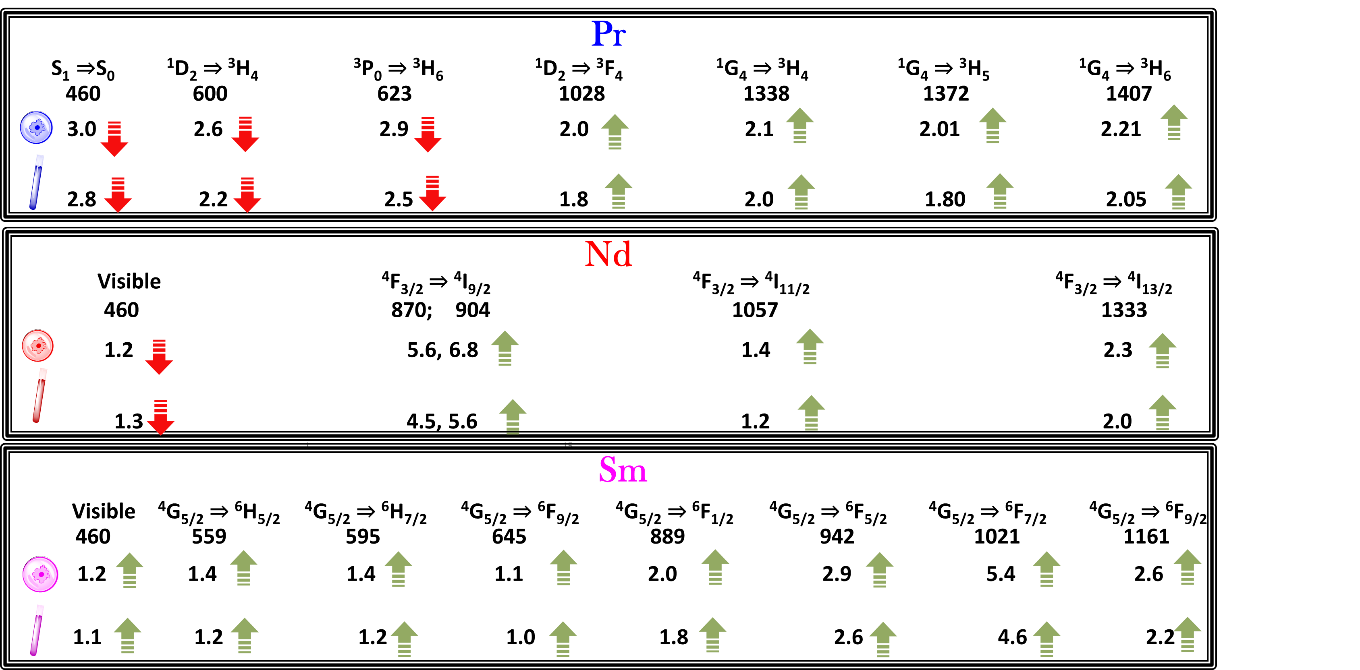 |

| 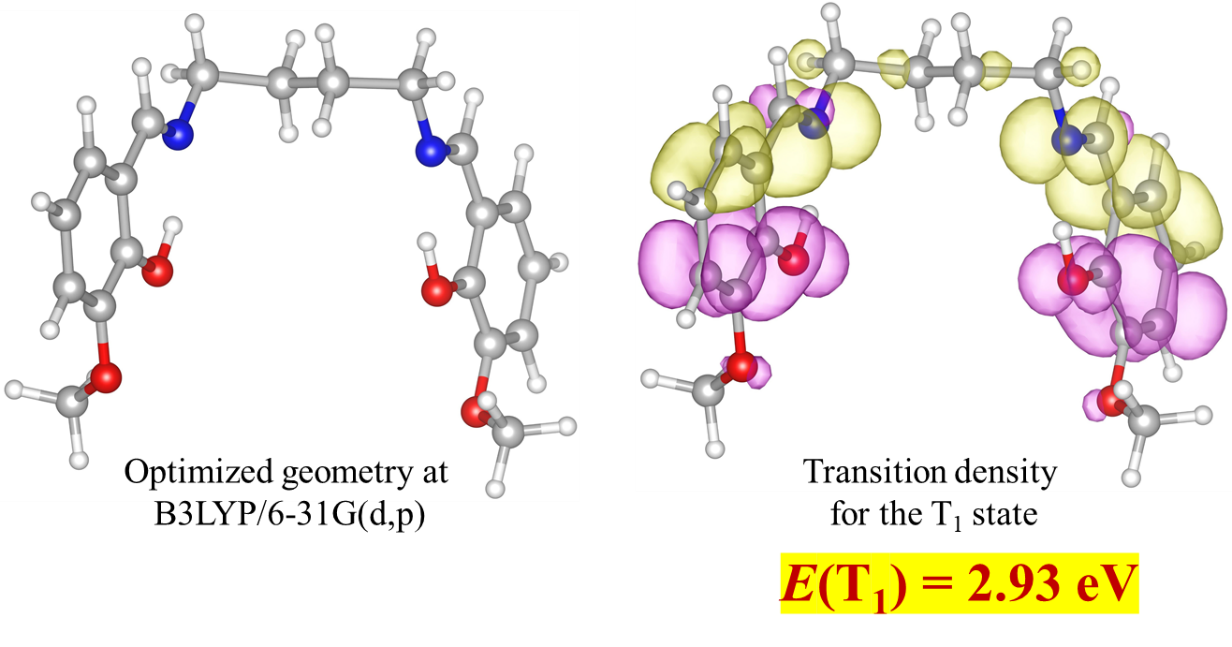 |
| --- |
| **Figure S8.** Triplet energy state calculation of ligand. |

| 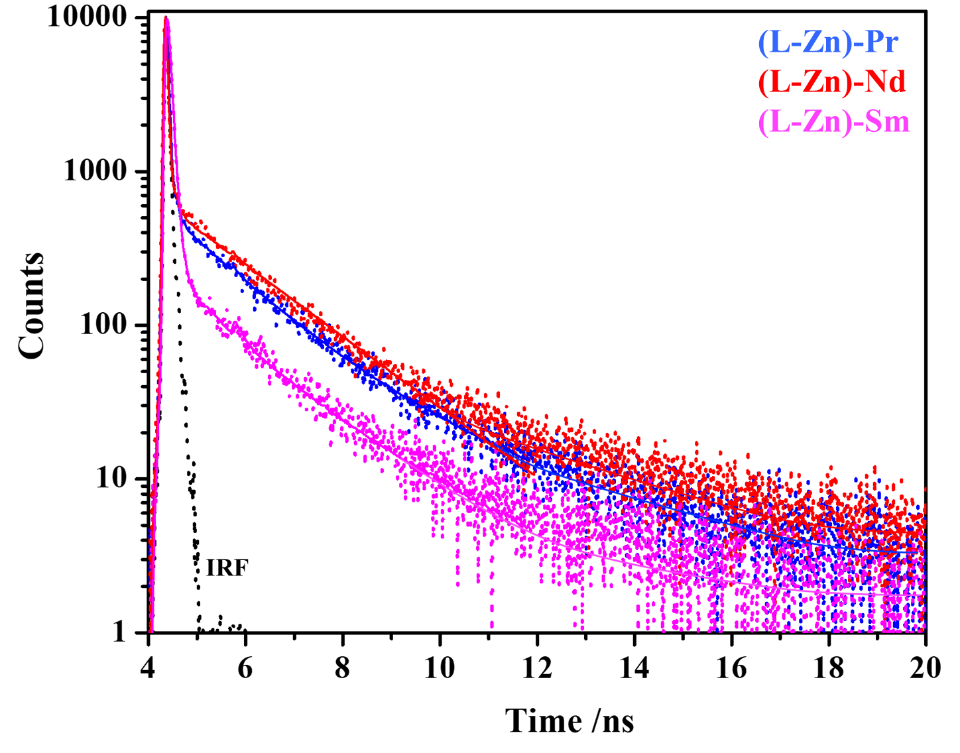 |
| --- |
| **Figure S9.** Luminescence decays of bimetallic lanthanide complexes in methanolic solution. The decays were obtained by exciting the solution with a 374 nm laser. The monitoring wavelength is 460 nm. The experiment was done using the facility available in Prof. Pratik Sen’s laboratory at IIT-Kanpur. |

**Reference**:

1. Armarego, W.; Perin, D. D. Purification of Laboratory Chemicals, 4th ed., **1996**.
2. Sheldrick, G. M. Crystal Structure Refinement with Shelxl. *Acta Crystallographica Section C Structural Chemistry* **2015**, 71 (1), 3–8.
3. Spackman, M. A.; Byrom, P. G. *Chem. Phys. Lett.* 1997, 267, 215. (b) McKinnon, J. J.; Mitchell, A. S.; Spackman, M. A*. Chem. Eur. J.* **1998**, 4, 2136.
4. Chilton, N. F.; Anderson, R. P.; Turner, L. D.; Soncini, A.; Murray, K. S. PHI: A Powerful New Program for the Analysis of Anisotropic Monomeric and Exchange-Coupled Polynuclear d- and f block Complexes. *J. Comput. Chem.* **2013**, 34, 1164−1175.
5. Dolomanov, O. V.; Bourhis, L. J.; Gildea, R. J.; Howard, J. A. K.; Puschmann, H. OLEX2: A Complete Structure Solution, Refinement and Analysis Program*. J. Appl. Crystallogr.* **2009**, 42, 339−341.
6. Sheldrick, G. M. Crystal Structure Refinement with SHELXL. *ActaCrystallogr., Sect. C: Struct. Chem.* **2015**, 71, 3−8.
